# Time-resolved transcriptome analysis reveals molecular signatures of *Fusarium proliferatum* DSM106835-induced sudden decline syndrome in date palm (*Phoenix dactylifera* L.)

**DOI:** 10.1093/jxb/eraf540

**Published:** 2025-12-16

**Authors:** Gouthaman P Purayil, Khaled A El-Tarabily, Frank M You, Synan F AbuQamar

**Affiliations:** Department of Biology, College of Science, United Arab Emirates University, Al Ain 15551, United Arab Emirates; Department of Biology, College of Science, United Arab Emirates University, Al Ain 15551, United Arab Emirates; Ottawa Research and Development Centre, Agriculture and Agri-Food Canada, Ottawa, ON K1A 0C6, Canada; Department of Biology, College of Science, United Arab Emirates University, Al Ain 15551, United Arab Emirates; University of Ghent, Belgium

**Keywords:** Co-expression networks, date palm, *Fusarium proliferatum*, hormone signaling, MAPK signaling, plant immunity, RNA-seq

## Abstract

The strategic Middle Eastern crop date palm is severely threatened by *Fusarium proliferatum* DSM106835 (*Fp*), the fungus causing sudden decline syndrome. To decipher the molecular basis of this interaction, we performed a time-series RNA-seq analysis to elucidate the dynamic transcriptomic responses in date palm roots and shoots to *Fp* infection at 4, 8, and 16 days post-infection (dpi). Thousands of genes showed altered expression, increasing dramatically over time: 4062 and 2741 differentially expressed genes in roots and shoots, respectively, at 4 dpi, rising to 10 670 and 4781 at 8 dpi, and 19 092 and 8570 by 16 dpi. The infection activated core defense pathways, including pathogen-triggered immunity and effector-triggered immunity, and key responses involved reactive oxygen species accumulation, cell wall remodeling, impaired photosynthesis, and reprogramming of hormone signaling pathways for ethylene, jasmonic acid, abscisic acid, and salicylic acid. Changes occurred in primary and secondary metabolism, covering carbohydrates, amino acids, lipids, and phenylpropanoids. Weighted gene co-expression network analysis identified tissue-specific gene modules and critical hub genes associated with *Fp* responses. This comprehensive analysis provides novel insights into date palm defense mechanisms against *Fp* infection. The identified key pathways and genes form a crucial foundation for targeted breeding or biocontrol strategies to enhance resistance against sudden decline syndrome.

## Teaser

To combat sudden decline syndrome caused by *Fusarium proliferatum* DSM106835, date palm activates key defense genes, hormones, and metabolism changes over time.

## Introduction

Date palm (*Phoenix dactylifera* L.) is a crucial crop in arid and semi-arid regions, particularly in the Middle East and North Africa, where it plays a vital role in food security and economic stability. Dates provide essential carbohydrates, fiber, minerals, and antioxidants (Ortiz-Uribe *et al.*, 2019). Although date palm is naturally resilient to desert conditions (Al-Khayri *et al.*, 2015), optimal yield requires intensive management and irrigation (Al-Omran *et al.*, 2019). In the United Arab Emirates (UAE), date palm is agriculturally and culturally significant. However, climate change and fungal pathogens, notably the *Fusarium fujikuroi* species complex (FFSC), threaten production (Savary *et al.*, 2019; Yilmaz *et al.*, 2021). *Fusarium proliferatum* DSM106835 (*Fp*) is a primary cause of sudden decline syndrome (SDS), a devastating disease characterized by root rot, wilting, and major economic losses (Purayil *et al.*, 2023).

SDS is a complex soil-borne disease caused by various pathogens, including *Fusarium* species (Abdalla, 2001; Jafari and Taghavi, 2007; Abedinzadeh *et al.*, 2023). In the UAE, *F. solani*, *F. proliferatum*, and *F. oxysporum* are the most aggressive SDS pathogens (Alwahshi *et al.*, 2019), with *F. proliferatum* becoming dominant recently (Purayil *et al.*, 2023). While agronomic and chemical practices exist for SDS management, the lack of identified resistance genes in date palm limits effective control strategies. Sustainable approaches, such as biological control agents like rhizospheric and endophytic actinobacteria, offer eco-friendly alternatives against *Fusarium* spp. (Saeed *et al.*, 2017; Alblooshi *et al.*, 2021; Alwahshi *et al.*, 2022; Galli *et al.*, 2024). Understanding plant–pathogen molecular interactions is key for long-term resistance.

Transcriptomics deciphers host molecular responses to pathogens. Comparative studies on *Rhizoctonia cerealis* in wheat, *Phytophthora palmivora* in *Nicotiana benthamiana*, and *Fusarium solani* in *Amorphophallus muelleri* highlight key resistance-related genes and pathways (Evangelisti *et al.*, 2017; Geng *et al.*, 2022; Gao *et al.*, 2023). Time-series transcriptome analysis captures dynamic responses, as evidenced for powdery mildew resistance in Kentucky bluegrass (*Poa pratensis*) (Zhang *et al.*, 2022). Thus, elucidating SDS molecular mechanisms, identifying resistance genes, and screening for resistant cultivars are vital sustainable strategies for disease management. Although key genes and potential mechanisms of root and stem rot caused by *F. proliferatum* in maize have been reported (Sun *et al.*, 2024), research on date remains limited.

Plants deploy a multi-layered immune system, beginning with pattern-triggered immunity (PTI), activated by pathogen-associated molecular patterns (PAMPs), and effector-triggered immunity (ETI), triggered by specific pathogen effectors (Jones and Dangl, 2006; Ngou *et al.*, 2022; Yu *et al.*, 2024). These signaling cascades lead to defense responses such as cell wall reinforcement, reactive oxygen species (ROS) production, and extensive transcriptional reprogramming mediated by phytohormones including salicylic acid (SA), jasmonic acid (JA), ethylene (ET), and abscisic acid (ABA) (Pieterse *et al.*, 2012; Zhao and Dixon, 2014; Molina *et al.*, 2024). These hormones often interact with key transcription factors (TFs) such as MYBs, NACs, ERFs, and WRKYs to fine-tune the defense response (Berrocal-Lobo *et al.*, 2002; Liu *et al.*, 2015; AbuQamar *et al.*, 2017; Deng *et al.*, 2020; Javed and Gao, 2023). Typically, SA mediates resistance against biotrophic pathogens, whereas JA and ET are central to defense against necrotrophs (Thomma *et al.*, 1998; Mengiste *et al.*, 2009). Secondary metabolites such as lignins (strengthening cell walls; Lee *et al.*, 2019), flavonoids, and phenolics (possessing antifungal properties; Erb and Kliebenstein, 2020) also contribute to plant defense, by fortifying cell walls and directly inhibiting fungal growth.

In this study, we hypothesize that *Fusarium proliferatum* DSM106835 induces a coordinated, tissue-specific transcriptomic response in date palm evolving over time, reflecting both local and systemic defenses. To test this, we performed a comprehensive time-series transcriptomic analysis of Barhee cultivar infected with *Fp* DSM106835 at 4, 8, and 16 days post-infection (dpi), analysing root and shoot tissues. The study aims to: (i) characterize the dynamic transcriptional reprogramming across tissues and infection stages; (ii) identify key genes, TFs, kinases, and hormonal pathways associated with PTI/ETI; and (iii) uncover co-expressed gene modules and tissue-specific hub genes using weighted gene co-expression network analysis (WGCNA) to delineate defense networks. These findings provide critical insights into the molecular basis of SDS and potential targets for breeding or biotechnological interventions aiming to enhance date palm disease resistance.

## Materials and methods

### Isolation and culturing of *Fusarium proliferatum*

*Fusarium proliferatum* strain DSM106835 was originally isolated from date palm trees exhibiting symptoms of SDS in Al Wagan area of Al Ain, Abu Dhabi, UAE (Alwahshi *et al.*, 2019; Purayil *et al.*, 2023). The isolate was cultured and maintained on potato dextrose agar plates (PDA; Lab M Ltd, Heywood, UK) supplemented with 25 mg l^−1^ penicillin–streptomycin (Sigma-Aldrich, Taufkirchen, Germany) to prevent bacterial contamination. Cultures were incubated at 25 °C and sub-cultured every 14 d to ensure viability and sustained growth.

### Fungal inoculation and disease quantification in plants

Six-month-old Barhee date palm seedlings, grown in autoclaved soil, were obtained from Al Wathba Marionnet LLC tissue culture facility (Abu Dhabi, UAE). Following a 2-week acclimatization period to eliminate potential biotic or abiotic influences, seedlings were inoculated with *Fp* by applying a spore suspension (1.0×10^6^ spores ml^−1^) directly to the root zone. Disease progression in root and shoot tissues was assessed at 0, 2, 4, 8, 16, and 32 dpi, and a disease susceptibility index (DSI) was developed based on the temporal quantification of conidia in infected tissues (Purayil *et al.*, 2023). Briefly, all inoculated seedlings exhibiting symptoms of SDS were evaluated using a 0–5 scale, where 0=no visible symptoms; 1=1–10% necrosis, leaf whitening, or root rot; 2=11–25%; 3=26–50%; 4=51–75%; and 5=76–100% tissue damage (Purayil *et al.*, 2023). The DSI for each plant was calculated based on the proportion of symptomatic tissue. To estimate fungal proliferation *in planta*, the number of conidia in affected tissues was quantified (Alblooshi *et al.*, 2021; Purayil *et al.*, 2023). A known weight of symptomatic root or shoot tissue was homogenized in 5 ml of sterile distilled water, and the resulting suspension was thoroughly mixed. The conidial concentration was then determined using a haemocytometer under a light microscope. The mean values of DSI and conidia concentration were determined from three independent biological replicates, each producing comparable results.

*Fp* nuclear ribosomal internal transcribed spacer (*ITS*) primers used for fungal biomass quantification were designed based on the *ITS* sequence of *F. proliferatum* DSM106835 (MH055399) (Schoch *et al.*, 2012; Alwahshi *et al.*, 2019; Purayil *et al.*, 2023). Primer specificity was confirmed by performing BLAST analysis against the NCBI nucleotide database, which revealed exclusive alignment to *Fp* sequences. Quantitative real-time PCR (qRT-PCR) was performed relative to the date palm ubiquitin (*UBQ*) gene (Moon *et al.*, 2004) across three independent biological replicates. Primer sequences for both genes are listed in Supplementary Table S1.

### Tissue sampling and RNA extraction for RNA-seq

Tissue samples were collected at 4, 8, and 16 dpi, representing key stages of physiological response to infection. Root and shoot tissues were harvested separately, with three biological replicates for each time point. All samples were immediately flash-frozen in liquid nitrogen and ground into a fine powder using a pre-chilled mortar and pestle.

Total RNA was extracted from 100 mg of powdered tissue using the Maxwell^®^ RSC Plant RNA Kit (Promega, Madison, WI, USA) according to the manufacturer’s instructions. RNA concentration and purity were measured using a NanoDrop spectrophotometer (Thermo Fisher Scientific, Waltham, MA, USA). RNA integrity was assessed with an Agilent 2100 Bioanalyzer (Agilent Technologies, Santa Clara, CA, USA).

### Library preparation and RNA sequencing

The mRNA was isolated from total RNA using oligo d(T)_25_ magnetic beads (New England Biolabs, Ipswich, MA, USA). The purified mRNA was fragmented using NEBNext^®^ RNA Fragmentation Buffer (New England Biolabs), and first-strand cDNA was synthesized via random hexamer-primed reverse transcription, followed by second-strand cDNA synthesis. End repair and A-tailing were performed using A-Tailing Mix (New England Biolabs), and RNA Index Adapters (BGI Group, Shenzhen, China) were ligated to the fragments. The resulting cDNA library was amplified, purified using AMPure XP beads (Beckman Coulter, Brea, CA, USA), and eluted in buffer (BGI Group). Library quality and fragment size distribution were assessed using a Bioanalyzer 2100 (Agilent Technologies).

To prepare for sequencing, double-stranded PCR products were denatured and circularized using splint-oligo sequences to generate single-stranded circular DNA (ssCir DNA). These templates were amplified using phi29 polymerase (New England Biolabs) to form DNA nanoballs, which were then loaded onto the DNBSEQ-G400 platform (BGI Group) for high-throughput sequencing. On average, approximately 40 million paired-end reads (150 bp) were generated per sample, ranging from 35 million to 45 million reads across all libraries. After quality filtering, >90% of the clean reads were successfully mapped to the reference genome (GCA_009389715.1) (Hazzouri *et al.*, 2019).

### Quality control and RNA-seq analysis

Quality control was performed using FastQC (v0.12.1) (Andrews, 2023) with default settings. Read trimming and filtering were carried out using SOAPnuke (v2.1.6) (Chen *et al.*, 2018) with parameters (-l 20 -q 0.2 -n 0.05). Alignment to the date palm (*P. dactylifera* cv. Barhee) reference genome (GCA_009389715.1) (Hazzouri *et al.*, 2019) was performed with STAR (v2.7.10a) (Dobin *et al.*, 2013) using default settings as implemented in the nf-core/rnaseq (v3.14.0) pipeline (Di Tommaso *et al.*, 2017; Patel *et al.*, 2024).

Transcript quantification was performed with Salmon (v1.10.1) (Patro *et al.*, 2017) in mapping-based mode with default parameters and GC bias correction enabled. Initial quality metrics including alignment rate and quantification accuracy were summarized using MultiQC (Ewels *et al.*, 2016), with downstream analysis conducted using DEGreport (Pantano *et al.*, 2024). Differential expression analysis was carried out using DESeq2 (v1.40.2) (Love *et al.*, 2014) with default normalization and shrinkage estimation. A custom annotation database for *P. dactylifera* was constructed using AnnotationForge (Carlson and Pagès, 2024). Gene Ontology (GO) and Kyoto Encyclopedia of Genes and Genomes (KEGG) pathway enrichment analyses were performed with ClusterProfiler (v4.10.0) (Xu *et al.*, 2024) and STRINGdb (Szklarczyk *et al.*, 2023), while pathway visualizations were generated using Pathview (Luo and Brouwer, 2013).

### Validation of differentially expressed genes

To validate the RNA-seq expression profiles, qRT-PCR was performed on a subset of key differentially expressed genes (DEGs). Total RNA was from the same three independent biological replicates per condition used for RNA-seq as described above. Gene-specific primers were designed using Primer3 software (Untergasser *et al.*, 2012) with an amplificon of 80–150 bp, a melting temperature of 58–60 °C, and a GC content of 40–60%. Primer specificity was confirmed by BLAST analysis against the *Phoenix dactylifera* genome and by ensuring a single peak in the melting curve analysis. The list of primers used for qRT-PCR is provided in Supplementary Table S1.

qRT-PCR reactions were performed in 20 µl volumes using the GoTaq® 1-Step qRT-PCR System (Promega) on a CFX96 Touch Real-Time PCR Detection System (Bio-Rad Laboratories, Hercules, CA, USA). A melt curve analysis (65–95 °C, increment 0.5 °C) was performed at the end of each run to confirm primer specificity.

The date palm *Actin-2* gene (*LOC103720863*) was used as the internal reference control for normalization, as it demonstrated stable expression across all samples in our experimental conditions (tissues and infection time points). Relative gene expression levels were calculated using the comparative $2^{-\Delta \Delta C_{T}}$ method (Livak and Schmittgen, 2001). Statistical significance between treatment groups (infected vs. control) at each time point was determined using one-way analysis of variance (ANOVA) as described in the ‘Statistical analysis’ section. Data are presented as the mean relative expression±SD of three biological replicates, each measured with two technical replicates.

### Identification and characterization of transcription factors and kinases in response to *Fp* infection

To identify kinases and TFs among the DEGs, gene IDs from each time point were mapped to the *P. dactylifera* genome, and corresponding FASTA sequences were retrieved. These sequences were analysed using the iTAK tool (Zheng *et al.*, 2016), which predicts and classifies genes as kinases or TFs based on domain composition and conserved motifs.

### Weighted gene co-expression network analysis

WGCNA was conducted to identify gene modules correlated with experimental traits. Transcript abundance was quantified using Salmon (Patro *et al.*, 2017) and normalized with the variance stabilizing transformation (VST) in DESeq2 (Lin *et al.*, 2008; Love *et al.*, 2014), retaining 27 548 expressed genes after filtering. A soft-thresholding power of β=12 was selected to approximate scale-free topology.

A signed co-expression network was constructed using the blockwiseModules function, with modules defined based on topological overlap and hierarchical clustering. Closely related modules were identified based on a module eigengene dissimilarity threshold of 0.25 and merged using the mergeCloseModules function. This threshold was selected to ensure biologically meaningful clustering while minimizing redundancy among modules. Unassigned genes were grouped into the grey module.

Module–trait relationships were determined by correlating module eigengenes with binary-coded metadata (tissue type, treatment, and time points). Hub genes were identified based on module membership (kME), with the top 20 most connected genes in each module designated as hubs. Networks of hub genes were visualized in Cytoscape (v3.9.1) (Shannon *et al.*, 2003) employing default layout settings.

### Statistical analysis

All experiments were conducted using three independent biological replicates unless otherwise stated. Statistical analyses of RNA-seq data, including normalization, differential gene expression, and principal component analysis (PCA), were performed using the DESeq2 package in R (Love *et al.*, 2014). GO and KEGG enrichment analyses in ClusterProfiler (v4.10.0) (Xu *et al.*, 2024) were performed using the Benjamini–Hochberg adjusted *P*<0.05 (Benjamini and Hochberg, 1995). WGCNA analysis used soft-thresholding power β=12 and mergeCutHeight=0.25, and other parameters were kept at default values (Langfelder and Horvath, 2008). All bioinformatic analyses were conducted using standard/default parameters unless otherwise stated.

For qRT-PCR validation, relative gene expression levels were calculated using the $2^{-\Delta \Delta C_{T}}$ method (Livak and Schmittgen, 2001), and significance between treatment groups was determined using one-way ANOVA followed by Tukey’s post-hoc test in GraphPad Prism v9.0 (GraphPad Software, Boston, MA, USA). All data are presented as means±SD, and statistical significance was set at *P*<0.05.

## Results

### Infection and disease progression in date palm seedlings infected with *Fusarium proliferatum*

**Fig. 1.**
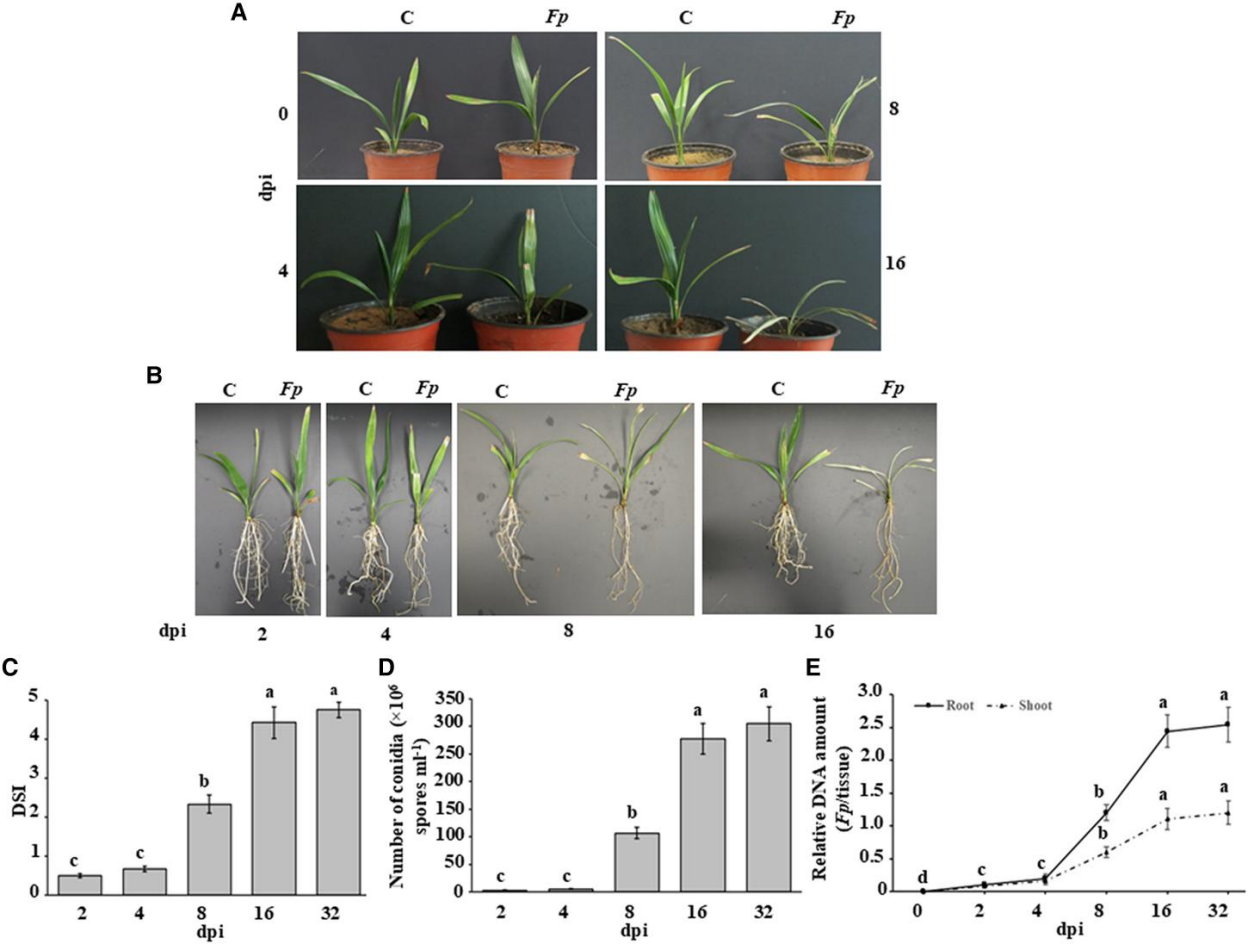
Disease progression in date palm tissues inoculated with *Fusarium proliferatum*. (A, B) Symptoms on shoot tissues (A) and disease development in roots (B) of date palm (cv. Barhi) seedlings inoculated with *Fp*. (C–E) DSI in shoot tissues (C), conidia counts in roots (D), and *Fp* biomass quantification (E) in inoculated plants. Disease progression was monitored post root-inoculation with *Fp* (1.0×10^6^ spores ml^−1^) at 0, 2, 4, 8, 16, and 32 dpi. In (A, B) photos were taken on the specified days. In (C) DSI was assessed on a 5-point scale: 0 (no infection) to 5 (76–100% damage, including necrosis or root rot). In (E), fungal biomass was measured by qRT-PCR, comparing *Fp ITS* relative to date palm *UBQ*. In (C–E) values (*n*=12) represent mean DSI, conidial counts in roots, and qRT-PCR results, respectively, from three independent replicates. Values with different letters are significantly different (*P*=0.05). C, control; *Fp*, *F. proliferatum*; dpi, days post-inoculation; DSI, disease severity index; *ITS*, internal transcribed spacer; *UBQ*, ubiquitin.

We characterized SDS progression in date palm seedlings using a time-course assessment of disease symptoms and fungal proliferation. Seedlings showed almost no visible symptoms at 4 dpi, suggesting an initial asymptomatic phase (Fig. 1A). By 8 dpi, leaf wilting marked the onset of visible SDS, which intensified by 16 dpi with severe wilting and noticeable root shrinkage (Fig. 1A, B). Disease progression, quantified by DSI and fungal conidia counts, increased steadily until 16 dpi (Fig. 1C, D). qRT-PCR analysis confirmed progressive fungal colonization, with a significant increase in *Fp* ITS gene amplification from 4 to 16 dpi in both root and shoot tissues (Fig. 1E). No significant differences in DSI, conidia counts, or fungal biomass were observed between 16 and 32 dpi (Fig. 1C, E). Based on these findings, we selected 4, 8, and 16 dpi as key time points for further transcriptomic analysis to capture early, transitional, and advanced stages of disease.

### Identification of differentially expressed genes

To elucidate the molecular responses of *Fp* infection, we performed transcriptome profiling across infected root and shoot tissues. RNA-seq reads were mapped to the date palm genome comprising 29 239 protein-coding genes of which 29 194 genes (Supplementary Table S2) showed non-zero read counts (Hazzouri *et al.*, 2019), and quality metrics confirmed data integrity (Supplementary Fig. S1). PCA revealed minimal variance among replicates, indicating high experimental consistency, and clear separation based on tissue and treatment, signifying transcriptional reprogramming (Supplementary Fig. S2).

**Fig. 2.**
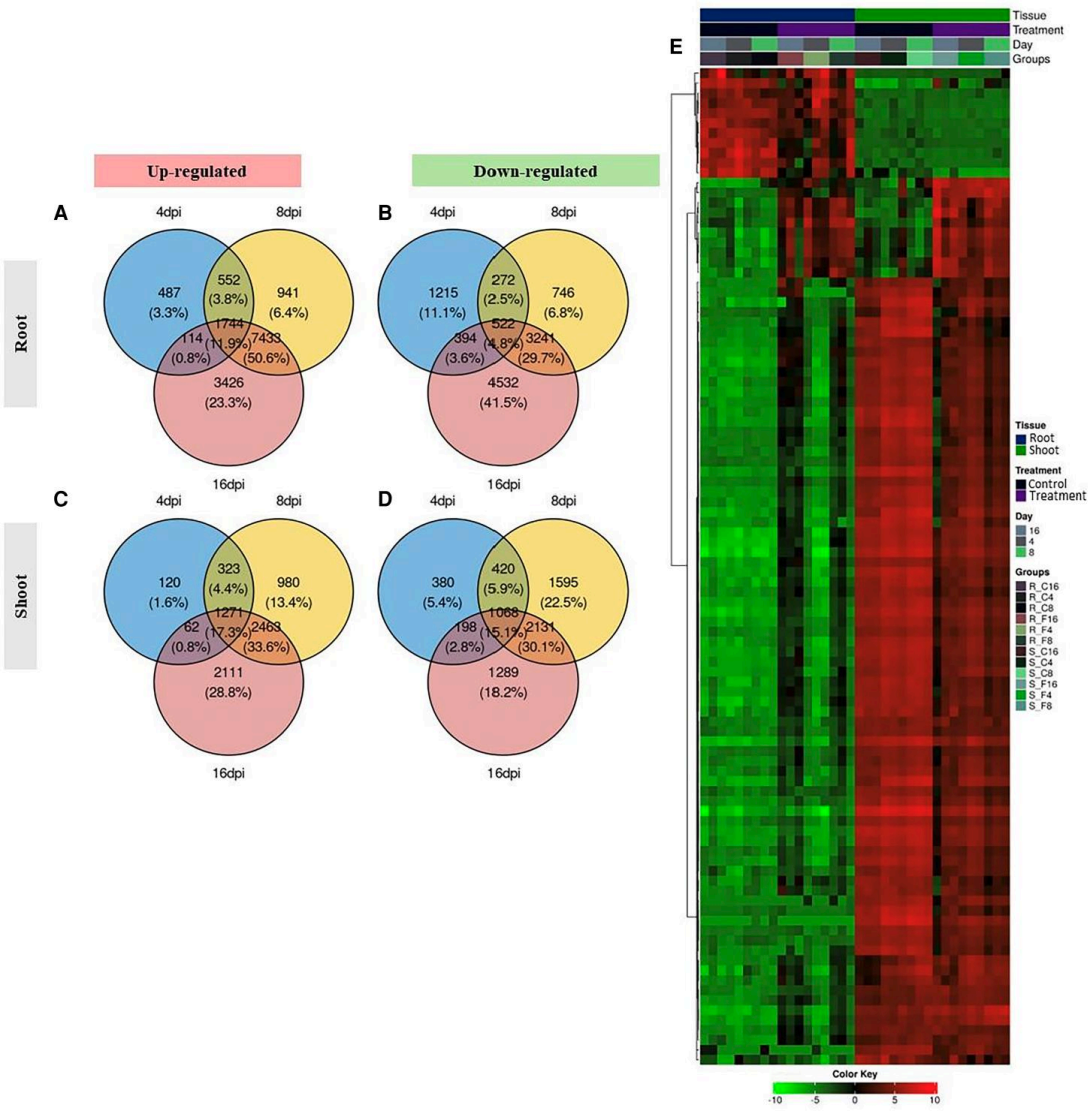
Differential gene expression in root and shoot samples in response to *Fusarium proliferatum* DSM106835 inoculation. (A–D) Venn diagram of up-regulated (A, C) and down-regulated (B, D) genes in root (A, B) and shoot (C, D) samples at 4, 8, and 16 dpi. (E) Heatmap showing gene expression patterns of highly differentially expressed genes (*P*-adjusted cutoff <0.05). Color intensity indicates gene expression levels, with red indicating up-regulation and green representing down-regulation. The groups being compared are 4, 8, and 16 dpi under control and disease conditions. Log-transformed expression levels, ranging from −10 to 10, were used to generate the heatmap. dpi, days post-inoculation.

A progressive, time-dependent increase in the number of DEGs was observed in both roots and shoots, with a more pronounced response in the directly infected root tissues (Fig. 2A–D). Specifically, 2897, 10 670, and 12 717 genes were up-regulated at 4, 8, and 16 dpi, respectively (Fig. 2A), while 2403, 4781, and 8689 were down-regulated at the same time points (Fig. 2B; Supplementary Table S3). Shoots showed a similar pattern but with fewer DEGs: 1776, 5037, and 5907 genes were up-regulated (Fig. 2C), and 2066, 5214, and 4686 were down-regulated at 4, 8, and 16 dpi, respectively (Fig. 2D; Supplementary Table S3). A global transcriptional heatmap reflected a pronounced tissue-specific shift (Fig. 2E), highlighting spatially discrete, stress-adaptive responses.

When applying an adjusted *P*-value threshold (<0.05), root tissues exhibited 2266 up-regulated genes and 1796 down-regulated genes at 4 dpi, 9620 up-regulated and 3851 down-regulated at 8 dpi, and 11 919 up-regulated and 7173 down-regulated at 16 dpi (Supplementary Fig. S3). In shoots, 1293 genes were up-regulated and 1448 were down-regulated at 4 dpi, followed by 4250 up-regulated and 4353 down-regulated at 8 dpi, and 5038 up-regulated and 3532 down-regulated at 16 dpi. Together, these data suggest distinct and dynamic transcriptional responses in roots and shoots to *Fp* infection.

### Gene ontology analysis of differentially expressed genes in response to *Fusarium proliferatum* infection

**Fig. 3.**
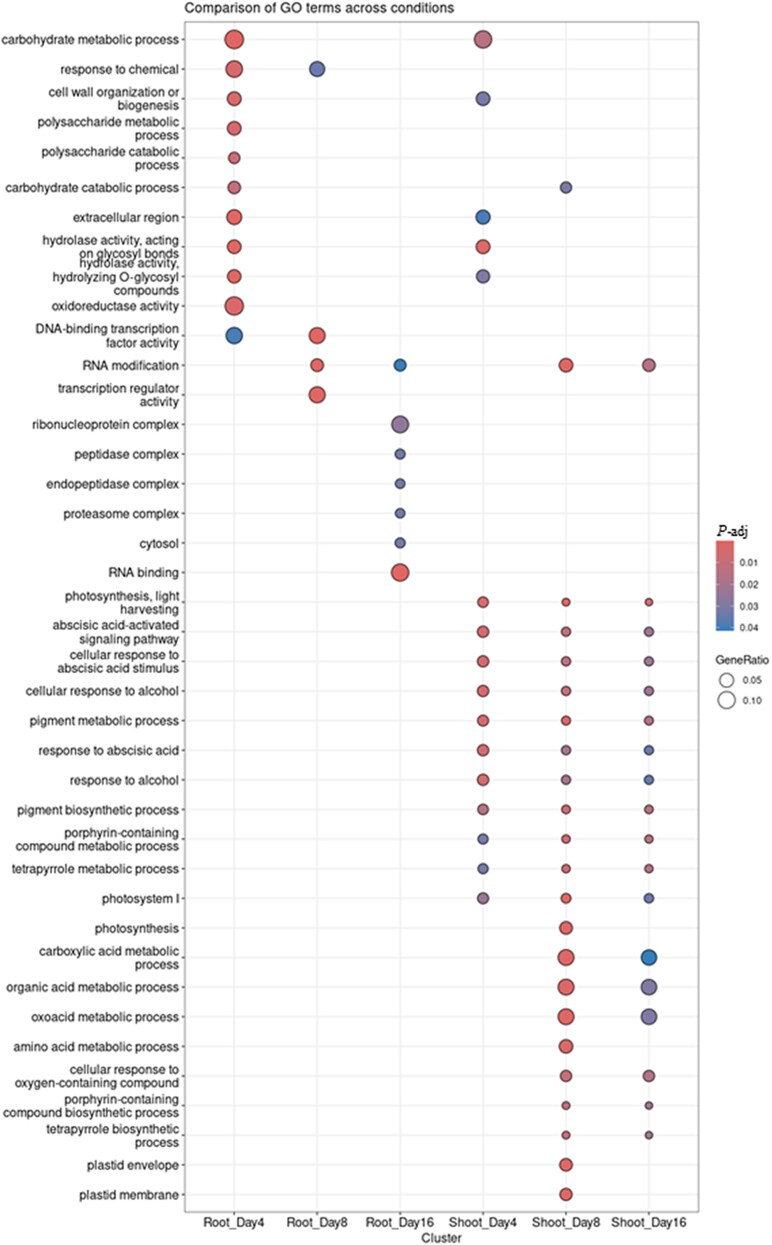
Temporal and tissue-specific Gene Ontology enrichment analysis of date palm in response to *Fusarium proliferatum* DSM106835 infection. The dot plot displays significantly enriched GO terms (biological processes, cellular components, and molecular functions) for DEGs in root and shoot tissues at 4, 8, and 16 dpi. The color intensity of the dots represents the statistical significance of the enrichment (log_2_FC≥1; adjusted *P*<0.05), and the dot size is proportional to the number of DEGs associated with each term. DEG, differentially expressed gene; dpi, days post-inoculation; FC, fold change; GO, Gene Ontology; *P-*adj, adjusted *P*-value.

GO enrichment analysis revealed distinct temporal shifts in the molecular functions of DEGs. In root tissues, early responses (4 dpi) were enriched for terms related to immune signaling and structural reinforcement, such as ‘response to chemical’, ‘cell wall organization’, and ‘DNA-binding transcription factor activity’ (Fig. 3). By 8 dpi, terms like ‘RNA modification’ and ‘transcription regulator activity’ were enriched, reflecting an ongoing response to the pathogen. At 16 dpi, enrichment shifted towards proteolytic components (‘proteosome complex’, ‘peptidase complex’), suggesting a transition to cellular degradation under prolonged stress.

In shoot tissues, early indicators of infection were linked to photosynthesis disturbance, with enrichment of terms like ‘photosystem I’ and ‘photosynthesis, light harvesting’, at 4 dpi (Fig. 3). Pathways for ‘abscisic acid activated signaling’ were also enriched, indicating the role of ABA in systemic signaling. This disruption continued at 8 dpi, accompanied by the initiation of organic acid metabolism. By 16 dpi, the expression patterns largely persisted, though enrichment for plastid-specific terms was no longer evident (Fig. 3).

### Kyoto Encyclopedia of Genes and Genomes pathway analysis during *Fp* infection

**Fig. 4.**
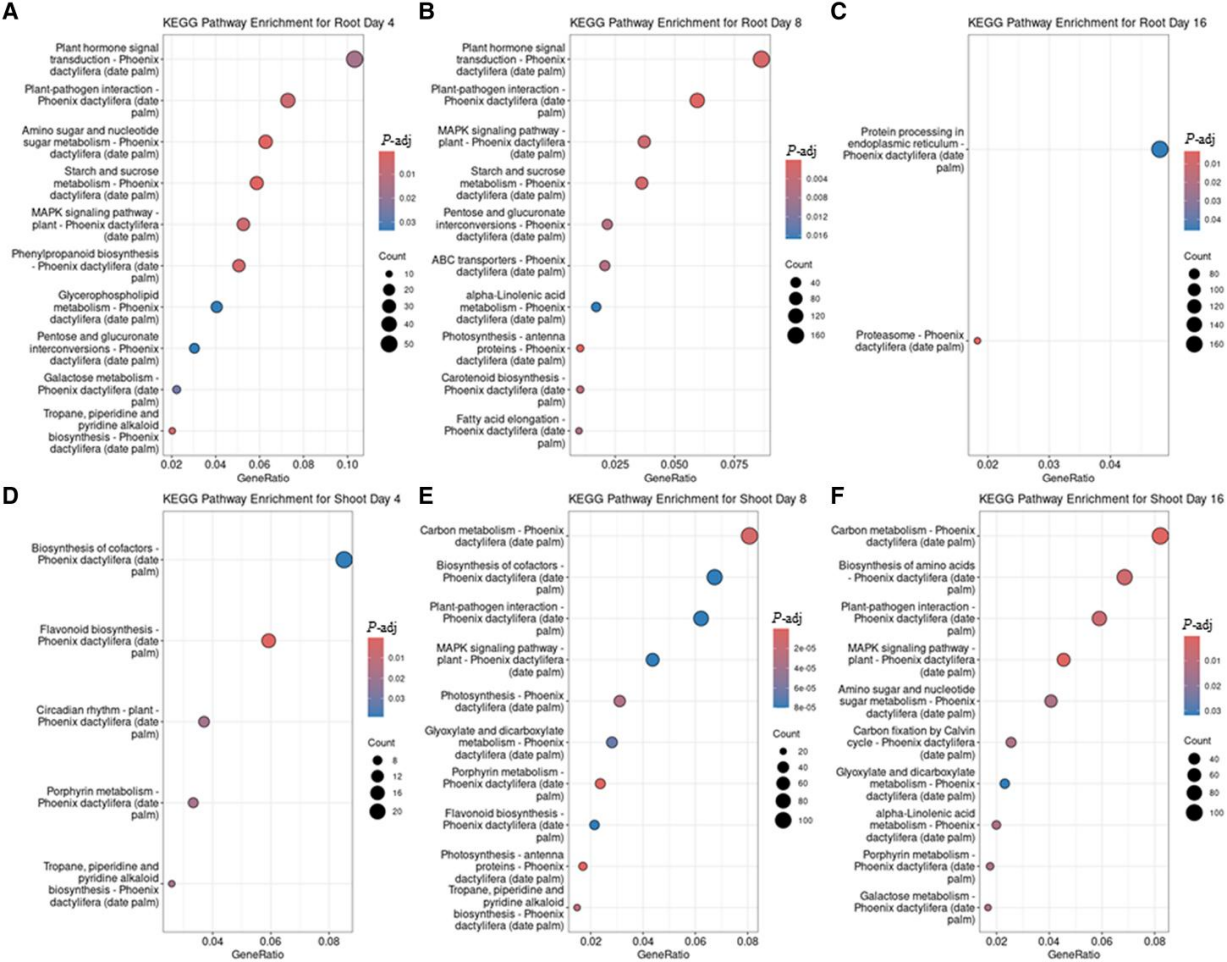
Dynamics of pathway activation in date palm roots and shoots during *Fusarium proliferatum* DSM106835 infection. KEGG pathway enrichment analysis of DEGs in root (A–C) and shoot (D–F) tissues at 4 dpi (A, D), 8 dpi (B, E), and 16 dpi (C, F). The chart illustrates the top enriched pathways, with circle size indicating the number of DEGs and color indicating the enrichment significance. DEG, differentially expressed gene; dpi, days post-inoculation; KEGG, Kyoto Encyclopedia of Genes and Genomes; *P-*adj, adjusted *P*-value.

KEGG pathway analysis identified significantly altered biological pathways. In roots at 4 dpi, we observed robust activation of defense-related pathways (Fig. 4A), including plant hormone signal transduction (94 genes), plant–pathogen interaction (55 genes), mitogen-activated protein kinase (MAPK) signaling (42 genes), and phenylpropanoid biosynthesis (40 genes; Supplementary Table S4). By 8 dpi, root tissues sustained strong defense responses with continued enrichment in these pathways (159 genes of plant–pathogen interaction, 201 of plant hormone signaling (Fig. 4B; Supplementary Table S4), and 48 genes of the ATP-binding cassette (ABC) transporter pathway. In contrast, by 16 dpi, defense-related enrichment declined markedly, with only proteasome-associated genes enriched (Fig. 4C; Supplementary Table S4).

In shoot tissues, early responses at 4 dpi involved the activation of secondary metabolism, including alkaloid (10 genes) and flavonoid (22 genes) biosynthesis (Fig. 4D; Supplementary Table S5), suggesting antioxidant and antimicrobial defenses. At 8 dpi, shoot defenses intensified with enrichment in 112 genes of the plant–pathogen interaction, and 72 genes of the MAPK signaling pathways, while 36 and 23 genes in porphyrin and photosynthesis pathways, respectively (Fig. 4E; Supplementary Table S5) indicated continued photosynthetic impairment. By 16 dpi, metabolic reprogramming became apparent, with enrichment in carbon metabolism (144 genes) and glyoxylate/dicarboxylate metabolism (43 genes) (Fig. 4F; Supplementary Table S5), indicating a shift toward energy generation. Sixty-three of the enriched genes in MAPK signaling remained active, suggesting ongoing defense activity.

### Response of the date palm seedlings to *Fusarium proliferatum* infection

**Fig. 5.**
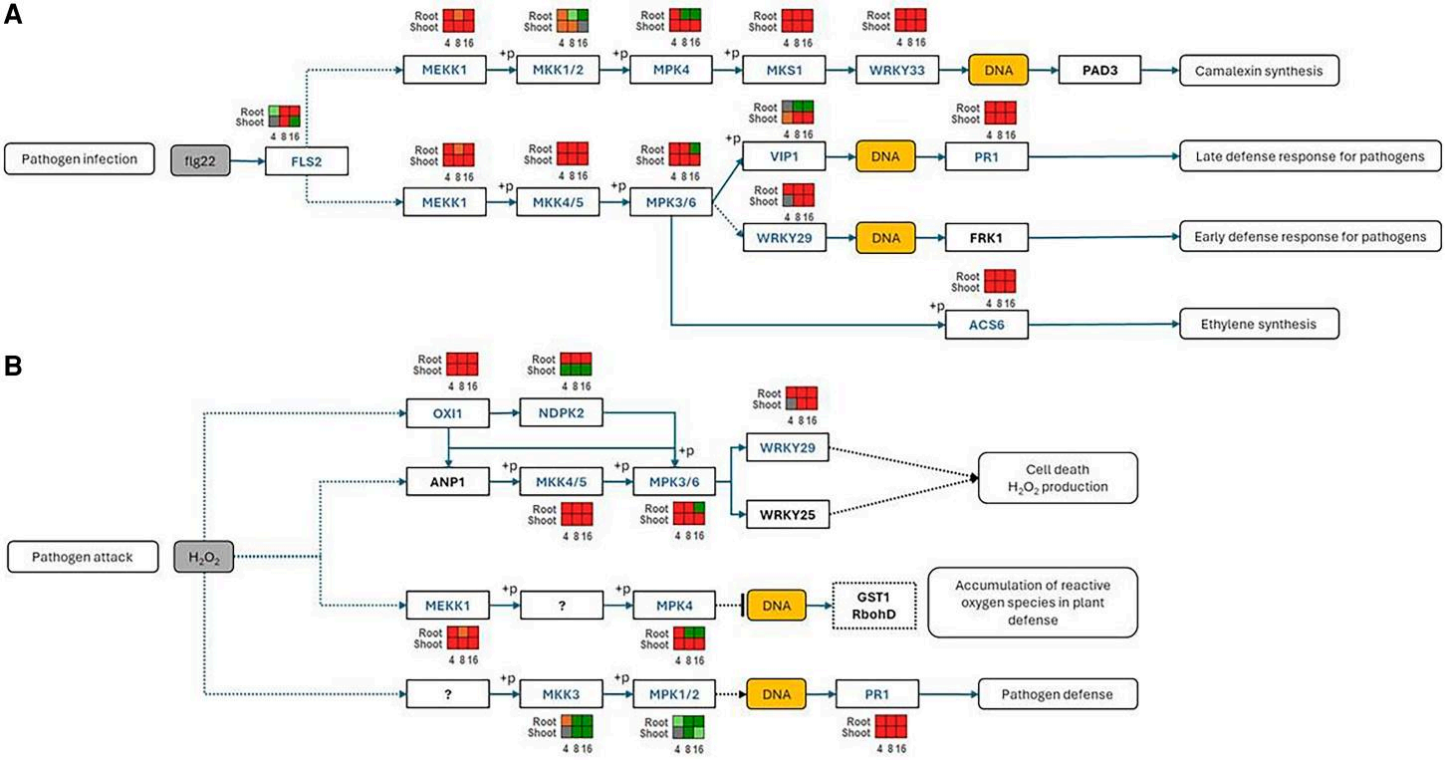
Transcriptional reprogramming of the MAPK (pda04016) signaling pathway in *Fusarium proliferatum* DSM106835-infected date palm. A simplified model of the MAPK cascade shows core components from pathogen recognition to downstream defense activation of FLS2 signaling (A) and oxidative stress signaling (B). Key DEGs are highlighted, with their expression changes (log_2_ fold change) indicated in adjacent heatmap tiles for root (R) and shoot (S) tissues at 4, 8, and 16 dpi. Red denotes up-regulation and green denotes and down-regulation. ACS6, 1-aminocyclopropane-1-carboxylic acid synthase 6; ANP1, and ortholog of the Arabidopsis NPK1-related protein kinase 1; DEG, differentially expressed gene; dpi, days post-inoculation; flg22, flagellin 22; FLS2, flagellin sensing 2; FRK1, FLG22-induced receptor-like kinase 1; GST1, glutathione *S*-transferase 1; H_2_O_2_, hydrogen peroxide; MAPK, mitogen-activated protein kinase; MEKK, MAPK kinase kinase; MKK, MAPK kinase; MKS, MAP kinase substrate 1; MPK, MAP kinase; NDPK2, nucleoside diphosphate kinase 2; OXI1, oxidative signal-inducible 1; PAD3, phytoalexin deficient 3; PR1, pathogenesis-related protein 1; RbohD, respiratory burst oxidase homologue D; VIP1, VIRE2-interacting protein 1.

Multilayered immune responses were evident, particularly in the MAPK signaling pathway (KEGG: pda04016), which transduces pathogen recognition signals into transcriptional reprogramming, with 163 DEGs across time points (Fig. 5A; Supplementary Table S6). Detection of the pathogen PAMP *flg22* by flagellin sensing 2 (FLS2) receptors (*LOC103721746*, *LOC103704912*) and ERECTA-like receptors (ERLs) initiated the MAPK cascade, involving consistent activation of MAP kinase kinase kinases (MAPKKKs; e.g. *LOC103715246*, *LOC103701811*, *LOC103703416*, *LOC103719487*) in both tissues. MAPK kinase kinase 1 (MEKK1; *LOC103715246*, *LOC103700827*) phosphorylation activated downstream MAP kinases (MPKs), regulating defense-related TFs.

Downstream, MPK4 (*LOC103707531*) activated WRKY33 (*LOC103708865*, *LOC103717512*, *LOC103721507*, *LOC108511557*), a regulator of camalexin biosynthesis, while MPK3 (*LOC103708987*) induced *1-aminocyclopropane-1-carboxylic acid* (*ACC*) *synthase 6* (*ACS6*; *LOC103699314*) to enhance ET production and activated WRKY29 (*LOC103709031*) for early defense gene expression (Fig. 5A). Phosphorylation of VIRE2-interacting protein 1 (VIP1; *LOC103701196*), together with the up-regulation of *pathogenesis-related protein 1* (*PR1*) genes (*LOC103712270*, *LOC103712272*, *LOC103713664*, *LOC103713666*), indicated activation of late defense.

A systematic oxidative burst, marked by hydrogen peroxide (H_2_O_2_) accumulation, was detected in roots (4 dpi) and in shoots (all time points) (Fig. 5B; Supplementary Table S6). This was mediated by MAPK kinase 4/5 (MKK4/5; *LOC103707100*, *LOC103718404*, *LOC103720430*) and nucleoside diphosphate kinase 2 (NDPK2; *LOC103714193*) activation, which up-regulated oxidative signal-inducible 1 (OXI1; *LOC103708546*, *LOC103717538*) to enhance *WRKY29* (*LOC103709031*) expression via MPK3/6 (*LOC103700827*, *LOC103708987*, *LOC103723061*) phosphorylation. This feedback loop reinforced early immunity (Fig. 5B). The number of DEGs in the MAPK pathway increased over time in both tissues (Supplementary Table S6). Root tissues showed 53, 73, and 62 up-regulated genes, and 9, 20, and 48 down-regulated genes, at 4, 8, and 16 dpi, respectively. In shoot tissues, 41, 60, and 69 genes were up-regulated, and 18, 23, and 25 genes were down-regulated at the same time points.

In the plant–pathogen interaction pathway (KEGG: pda04626), *chitin elicitor receptor kinase 1* (*CERK1*), a key pathogen recognition receptor (PRR), was significantly up-regulated (Supplementary Table S6), indicating sustained pathogen detection. The number of up- and down-regulated DEGs in this pathway also showed a progressive increase during infection. The number of up- and down-regulated DEGs in this pathway also progressively increased during infection. In roots, 89, 147, and 131 genes were up-regulated, while 21, 22, and 63 genes were down-regulated at 4, 8, 16 dpi, respectively. In shoots, 63, 110, and 106 genes were up-regulated, whereas 29, 33, and 36 genes were down-regulated at the corresponding time points.

### Impact of *Fusarium proliferatum* infection on metabolic pathways

**Fig. 6.**
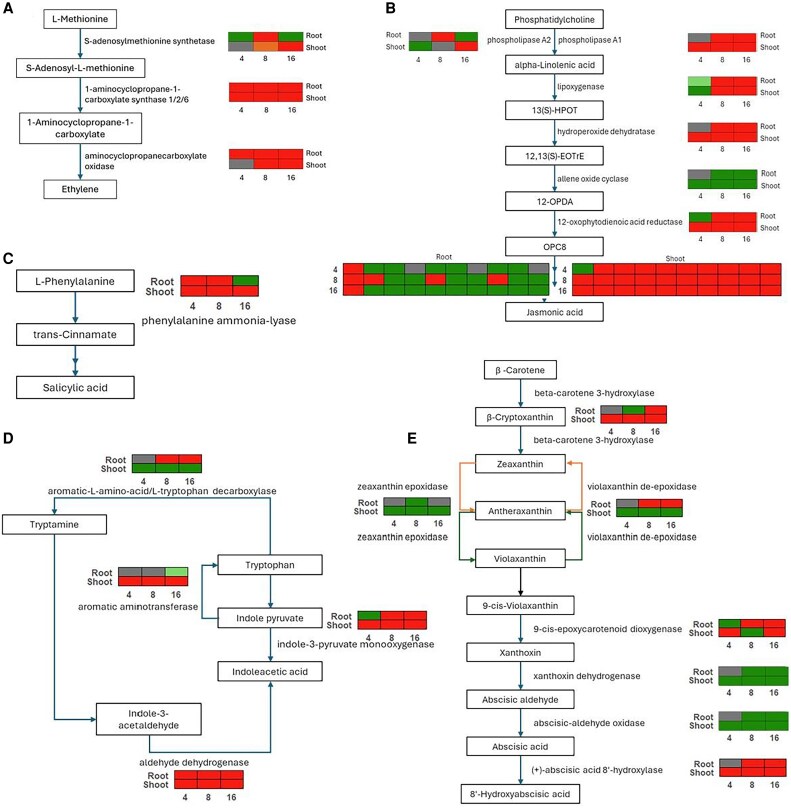
Hormone biosynthesis pathways are differentially regulated in date palm roots and shoots upon *Fusarium proliferatum* DSM106835 (*Fp*) challenge. Schematic depiction of the core biosynthetic pathways for ET (A), JA (B), SA (C), IAA (D), and ABA (E). Key enzymatic genes are shown, with their expression profiles (log2 fold change) displayed in adjacent heatmap tiles for root and shoot tissues at 4, 8, and 16 dpi with *Fp*. DEGs are color-coded based on log_2_ fold change values, where red and green represent up- and down-regulation, respectively. ET, JA, SA, IAA, and ABA pathways were constructed based on methionine (pda00270), α-linoleic acid (pda00592), phenylalanine (pda00360), tryptophan (pda00380), and carotenoid (pda00906) metabolism, respectively. 12-OPDA, 12-*oxo*-phytodienoic acid; 12,13(*S*)-EOTrE 12,13(*S*)-hydroperoxy-octadecatrienoic acid; 13(*S*)-HPOT, 13(*S*)-hydroperoxy-octadecatrienoic acid; ABA, abscisic acid; DEG, differentially expressed gene; dpi, days post-inoculation; ET, ethylene; IAA, indoleacetic acid; JA, jasmonic acid; OPC8, (9*S*,13*S*,15*Z*)-12-*oxo*-10,11-dihydrophyto-15-enoate; SA, salicylic acid.

Transcriptional changes in key metabolic pathways for hormone production were analysed. Cystine and methionine metabolism (pda00270; 136 genes) showed root up-regulation (31, 47, and 40 genes) and down-regulation (14, 24, and 48 genes) at 4, 8, and 16 dpi, respectively. Shoots had up-regulation (15/41/53 genes) and down-regulation (11/14/9 genes) for the same time points of infection. The key ethylene biosynthesis enzyme genes *ACC synthase* (*ACS*; *LOC103699314*) and *ACC oxidase* (*ACO*; *LOC103699725*, *LOC103713801*) were highly up-regulated in roots (Fig. 6A; Supplementary Table S7).

The α-linolenic acid metabolism pathway (pda00592; JA-linked) exhibited root up-regulation of 26, 36, and 27 genes and down-regulation of 7, 9, and 26 genes, and shoot up-regulation of 23, 36, and 35 genes and down-regulation of 9, 5, and 8 genes at 4, 8, and 16 dpi, respectively. Enzymes converting (9*S*,13*S*,15*Z*)-12-*oxo*-10,11-dihydrophyto-15-enoate (OPC8) to JA were generally more up-regulated in shoots (Fig. 6B; Supplementary Table S7), indicating tissue-specific JA modulation.

Expression of the gene for phenylalanine ammonia-lyase (*LOC103708643*, *LOC103708669*), which is crucial for SA precursor synthesis, in phenylalanine metabolism (pda00360) for the conversion of L-phenylalanine to *trans*-cinnamate, was induced in roots at 4 and 8 dpi and remained elevated in shoots (Fig. 6C; Supplementary Table S7). At 4, 8, and 16 dpi, 8, 12, and 10 genes were up-regulated and 7, 4, and 11 genes were down-regulated in root tissues, whereas 8, 10, and 11 genes were up-regulated and 2, 6, and 3 genes were down-regulated in shoot tissues, respectively.

Tryptophan metabolism (pda00380; IAA-linked) showed 24, 39, and 36 genes up-regulated and 8, 3, and 14 genes down-regulated in root tissue, while 15, 21, and 25 genes were up-regulated and 12, 19, and 11 genes were down-regulated in shoot tissues at 4, 8, and 16 dpi, respectively. Enzymes for converting tryptamine to indole-3-acetic acid (IAA) (*indole-3-pyruvate monooxygenase*: *LOC103697484*, *LOC103711336*, *LOC103723572*; and *aldehyde dehydrogenase*: *LOC103701633*, *LOC103710055*) were broadly up-regulated (Fig. 6D; Supplementary Table S7). Tryptophan metabolism (pda00380) showed root up-regulation (24, 39, and 36 genes, respectively) and down-regulation (8, 3, and 14 genes, respectively), with shoot up-regulation (15, 21, and 25 genes, respectively) and down-regulation (12, 19, and 11 genes, respectively) at 4, 8, and 16 dpi. Enzymes for converting tryptamine to IAA (*indole-3-pyruvate monooxygenase*: *LOC103697484*, *LOC103711336*, *LOC103723572*; *aldehyde dehydrogenase*: *LOC103701633*, *LOC103710055*) were broadly up-regulated.

The carotenoid biosynthesis pathway (pda00906) displayed significant changes: the gene for 9-*cis*-epoxycarotenoid dioxygenase (*LOC103705744*), converting 9-*cis*-violaxanthin to abscisic aldehyde (ABA precursor), was up-regulated (Fig. 6E). The gene for the ABA catabolism enzyme (*+*)-abscisic acid 8′-hydroxylase (*LOC103712508*) was up-regulated in both tissues, indicating dynamic ABA regulation. At 4, 8, and 16 dpi, a total of 12, 17, and 21 genes were up-regulated, while 5, 5, and 4 genes were down-regulated in roots, respectively. In shoots, 10 genes were consistently up-regulated across all time points, whereas 6, 12, and 9 genes were down-regulated at 4, 8, and 16 dpi, respectively (Fig. 6E; Supplementary Table S7).

### Hormone signaling dynamics in response to *Fusarium proliferatum* infection

**Fig. 7.**
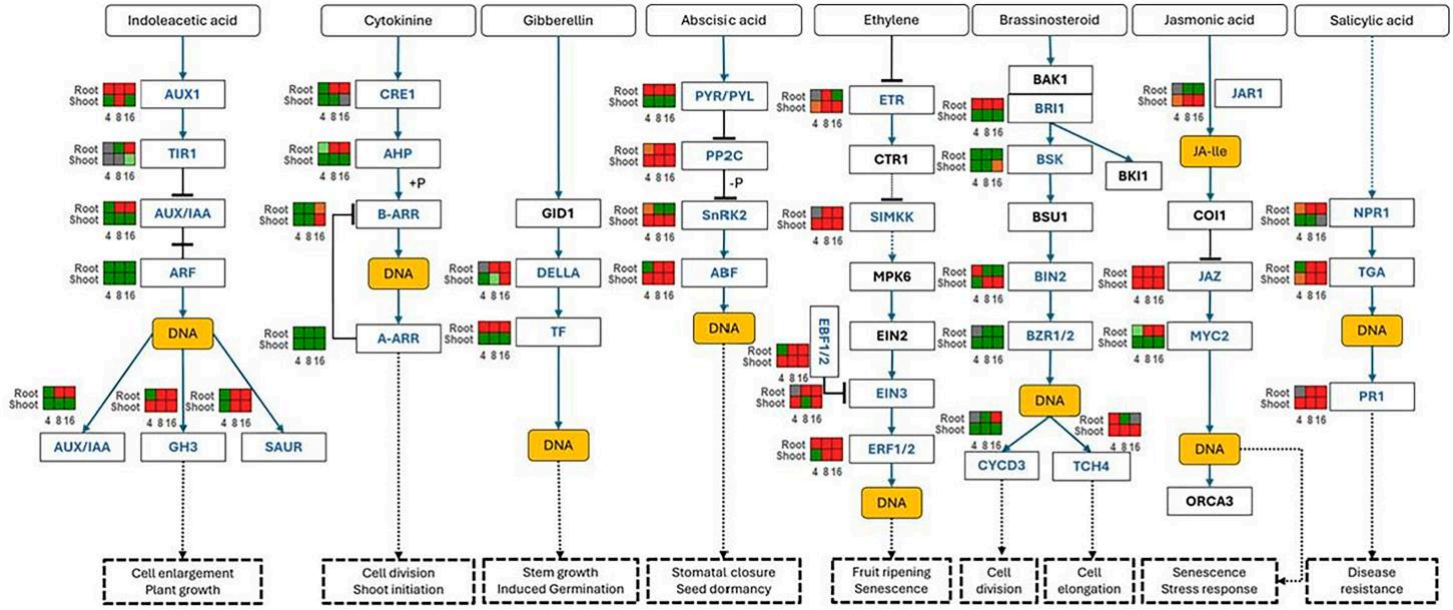
Analysis of plant hormone signal transduction pathway (pda04075) in tissues of date palm plants inoculated with *Fusarium proliferatum* DSM106835. Scheme representing the involvement of DEGs in hormone signal transduction pathway of indoleacetic acid, cytokinin, gibberellin, abscisic acid, ethylene, brassinosteroid, jasmonic acid, and salicylic acid, in root and shoot tissues of date palm plants at 4, 8, and 16 dpi with *F. proliferatum*. Each heatmap represents the DEG from respective pathways for a specific condition. DEGs are color-coded based on log_2_ fold change values, where red and green represent up- and down-regulation, respectively. ABF, ABRE-binding factor; AHP, histidine phosphotransfer protein; ARF, auxin response factor; AUX1, auxin influx carrier; AUX/IAA, auxin/indole-3-acetic acid; ARR, Arabidopsis response regulator; BAK1, BRI1-associated receptor kinase 1; BIK1, *Botrytis*-induced kinase 1; BIN2, brassinosteroid-insensitive 2; BRI1, brassinosteroid-insensitive 1; BSK, brassinosteroid-signaling kinase; BSU, brassinolide-insensitive 1 suppressor 1; BZR, brassinazole resistant; COI1, coronatine insensitive 1; CRE1, cytokinin response 1; CTR1, constitutive triple-response 1; CYCD3, cyclin D3; EIN, ethylene insensitive; ERF, ethylene responsive factor; ETR, ethylene receptor; GH3, Gretchen Hagen 3; GID1, gibberellin insensitive dwarf 1; JA-Ile, jasmonoyl isoleucine; JAR1, jasmonate resistant 1; JAZ, jasmonate ZIM-domain; MKK, MAPK kinase; MPK6, MAP kinase 6; MYC2, myelocytomatosis; NPR1, nonexpressor of pathogenesis-related 1; ORCA4, octadecanoid-Responsive *Catharanthus* AP2-domain 4; PP2C, protein phosphatase type 2C; PR1, pathogenesis-related protein 1; PYR/PYL, pyrabactin resistance receptor; SAUR, small auxin-up-regulated RNA; SnRK2, snf1-related protein kinase 2; TCH4, touch 4; TGA, TGACG motif-binding factor; TF, transcription factor; TIR1, toll/interleukin-1 receptor; DEG, differentially expressed gene; dpi, days post-inoculation.

We examined the transcriptional regulation of major signaling pathways (Fig. 7). A substantial number of hormone-related genes were differentially expressed, showing dynamic and tissue-specific regulation. In roots, 220, 233, and 226 genes were up-regulated, while 201, 189, and 197 were down-regulated at 4, 8, and 16 dpi, respectively. In shoots, 195, 224, and 215 genes were up-regulated, and 215, 189, and 198 down-regulated at the corresponding time points (Supplementary Table S8).

Auxin (AUX) signaling in roots featured consistent up-regulation of *auxin influx carrier* (*AUX1*; *LOC120108273*), but auxin response factor (ARF) down-regulation. *Small auxin-up-regulated RNA* (*SAUR*) (*LOC103712188*, *LOC103713151*, *LOC103713388*), *AUX/IAA* (*LOC103720508*, *LOC103721888*), and *auxin-responsive Gretchen Hagen3* (*GH3*) (*LOC103699533*, *LOC103700908*, *LOC120108159*, *LOC120113231*) components were up-regulated throughout, suggesting stress-related growth adjustment. In shoots, *AUX/IAA* (*LOC103702546*), *AUX1* (*LOC103701258*), and *ARF* were down-regulated, while *GH3* (*LOC120108159*, *LOC120113231*) and *SAUR* (*LOC103712188*, *LOC103713151*, *LOC103713388*) remained up-regulated (Supplementary Table S8), indicating delayed tissue-specific responses.

In roots, cytokinin (CK) signaling showed early suppression of expression of *cytokinin response 1* (*CRE1*; *LOC103696690*) and *histidine phosphotransfer proteins* (*AHP*; *LOC103696281*, *LOC103715395*) at 4 dpi, followed by up-regulation at 8 dpi. Response regulators (ARRs) were up-regulated only at 16 dpi. Shoots remained largely repressed, with limited ARR (*LOC103717294*, *LOC103723950*) activation at 16 dpi (Supplementary Table S8). Gibberellin (GA) signaling involved consistent up-regulation of *DELLA* (*LOC120103830*) repressors in roots, while in shoots, this emerged only at 16 dpi.

ABA signaling was initiated via *pyrabactin resistance receptor* (*PYR/PYL*; *LOC103706891*) and *snf1-related protein kinase* (*SnRK2*; *LOC103703209*) up-regulation in roots at 4 dpi (Supplementary Table S8). The negative regulator *PP2C* (*LOC103710370*, *LOC103715479*) induced at 8 dpi, potentially dampened the response. In shoots, *PYR/PYL* (*LOC103717496*, *LOC120112721*) was down-regulated, while *PP2C* (*LOC103697366*, *LOC103703044*) and *SnRK2* (*LOC103701777*, *LOC103703209*) up-regulation facilitated partial ABA signaling and ABRE-binding factor (ABF) activation.

ET signaling was strongly activated: ET receptors were up-regulated at 8 dpi in roots and continuously in shoots (Supplementary Table S8). Downstream *EIN2* (*LOC103705734*), *EIN3* (*LOC103709119*, *LOC103709120*), and *ERF1/2* (*LOC103703576*, *LOC103707018*) were consistently up-regulated in both tissues, alongside *EBF1/2* (*LOC120104346*), indicating robust transcriptional reprogramming.

Brassinosteroid (BR) signaling was initiated via *BRI1* (*LOC103720660*) up-regulation in roots but showed suppression in shoots (Supplementary Table S8). The negative regulator *BIN2* (*LOC103707356*) was repressed in roots but activated in shoots from 8 dpi, correlating with down-regulation of *BZR1* (*LOC103711311*, *LOC120107791*).

JA signaling featured up-regulation of *jasmonate-ZIM domain* (*JAZ*; *LOC103696392*) repressors in both tissues. Central activator *MYC2* (*LOC103706364*) was up-regulated only in roots at 8 and 16 dpi, indicating tight temporal control (Supplementary Table S8).

SA signaling began with *NPR1* (*LOC103714656*, *LOC103720717*) up-regulation in roots throughout, but down-regulation in shoots (Supplementary Table S8). Downstream *TGA* TFs (*LOC103705377*, *LOC103706198*) were broadly induced (except roots at 4 dpi), supporting SA-mediated defense.

### Identification of major kinase and transcription factor families

**Fig. 8.**
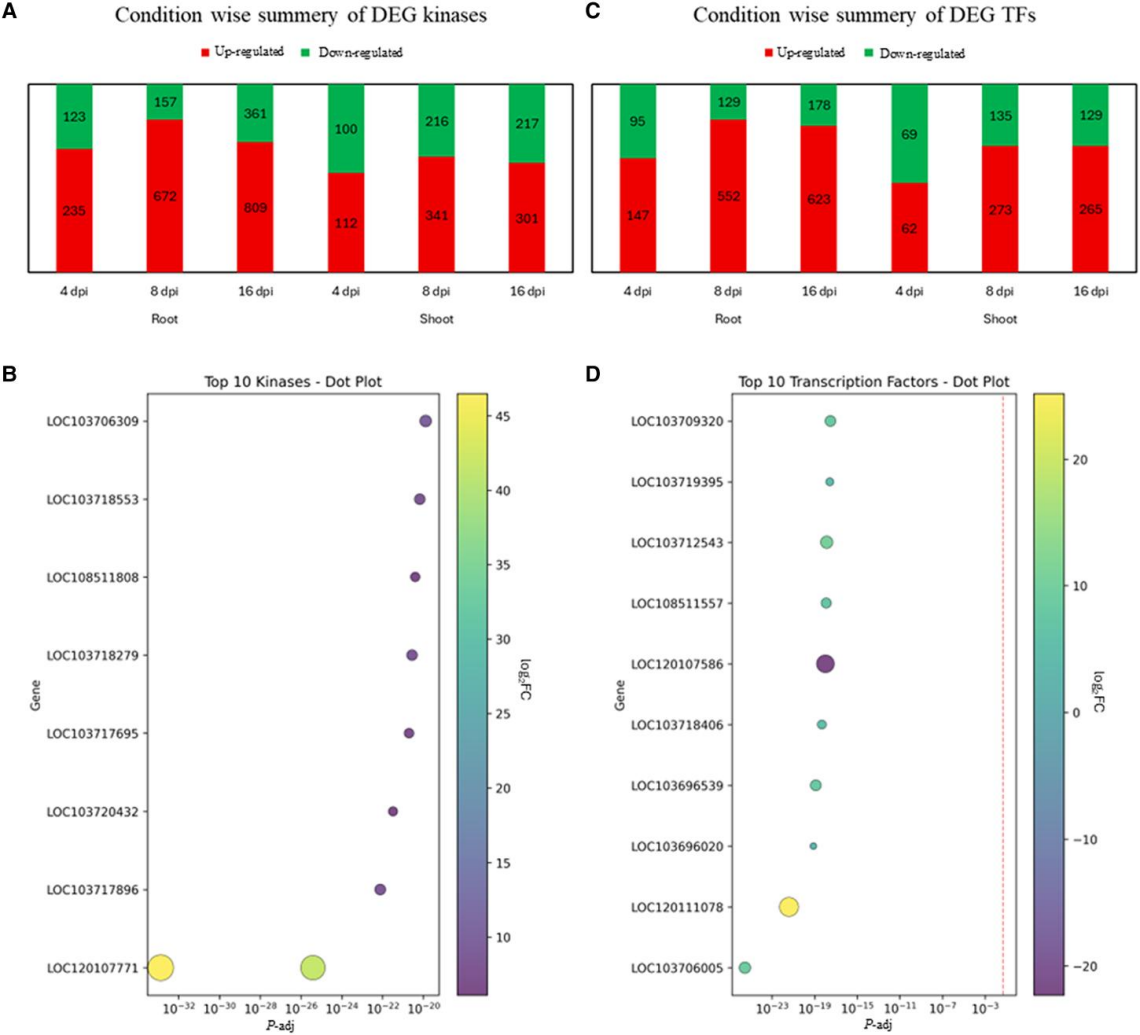
Kinase and transcription factor families show dynamic, tissue-specific responses to *Fusarium proliferatum* DSM106835. (A) Stacked bar chart showing the number of differentially expressed kinases in root and shoot tissues at 4, 8, and 16 dpi. (B) Dot plot of the top 10 most significantly differentially expressed kinases across all samples. (C) Stacked bar chart summarizing the number of differentially expressed TFs in root and shoot tissues across the three time points. (D) Dot plot of the top 10 most differentially expressed TFs. The *x*-axis indicates the adjusted *P*-value (*P-*adj), and the color represents the log_2_ fold change (log_2_FC). In (A, C), up-regulated genes are shown in red, and down-regulated genes are shown in green. In (B, D), dot color represents the average log_2_FC (red: up, blue: down), and the size represents the statistical significance (adjusted *P*<0.05). DEG, differentially expressed gene; dpi, days post-inoculation; TF, transcription factor.

Analysis of kinase families revealed a temporal escalation in kinase-mediated signaling, with 3644 significantly differentially expressed kinase genes identified across tissues. The number of differentially expressed kinases increased progressively with infection duration, reaching 358, 829, and 1170 in roots and 212, 557, and 518 in shoots at 4, 8, and 16 dpi, respectively (Fig. 8A). Root tissues exhibited a stronger response overall, with 2357 differentially expressed kinases compared to 1287 in shoots. The most represented families included RLK-Pelle_DLSV (333 instances), RLK-Pelle_LRR-XI-1 (239), and RLK-Pelle_SD-2b (157), while a *wall-associated kinase* (*WAK*) family member (*LOC120107771*) showed the highest up-regulation in roots (Fig. 8B; Supplementary Table S9). Moreover, members of the LRR and DLSV kinase families displayed pronounced differential expression patterns, highlighting their potential role in pathogen recognition and downstream defense signaling during *Fp* infection.

A total of 2657 differentially expressed TFs were identified, with a notable predominance of up-regulation (72.3%). The number of DETFs increased 3.2-fold from early (4 dpi; 373 genes) to late (16 dpi; 1195 genes) infection, with a more robust response in roots, accounting for 64.9% (1724 genes) compared to 35.1% (933 genes) in shoots (Fig. 8C). Among the top families, *ERF*, *NAC*, *C2H2*, *WRKY*, and *GRAS* showed the most pronounced activation (>75% of their members being up-regulated), while the MYB-related family had the highest number of down-regulated TFs in shoots at 4 dpi (Fig. 8D; Supplementary Table S10).

### Key gene modules activated by date palm seedlings during *Fusarium proliferatum* infection based on weighted gene co-expression network analysis

To uncover gene networks responsive to *Fp* infection in date palm seedlings, WGCNA was employed. This method enables the identification of clusters (modules) of highly correlated genes that may function together in biological processes such as defense responses and tissue-specific regulation.

**Fig. 9.**
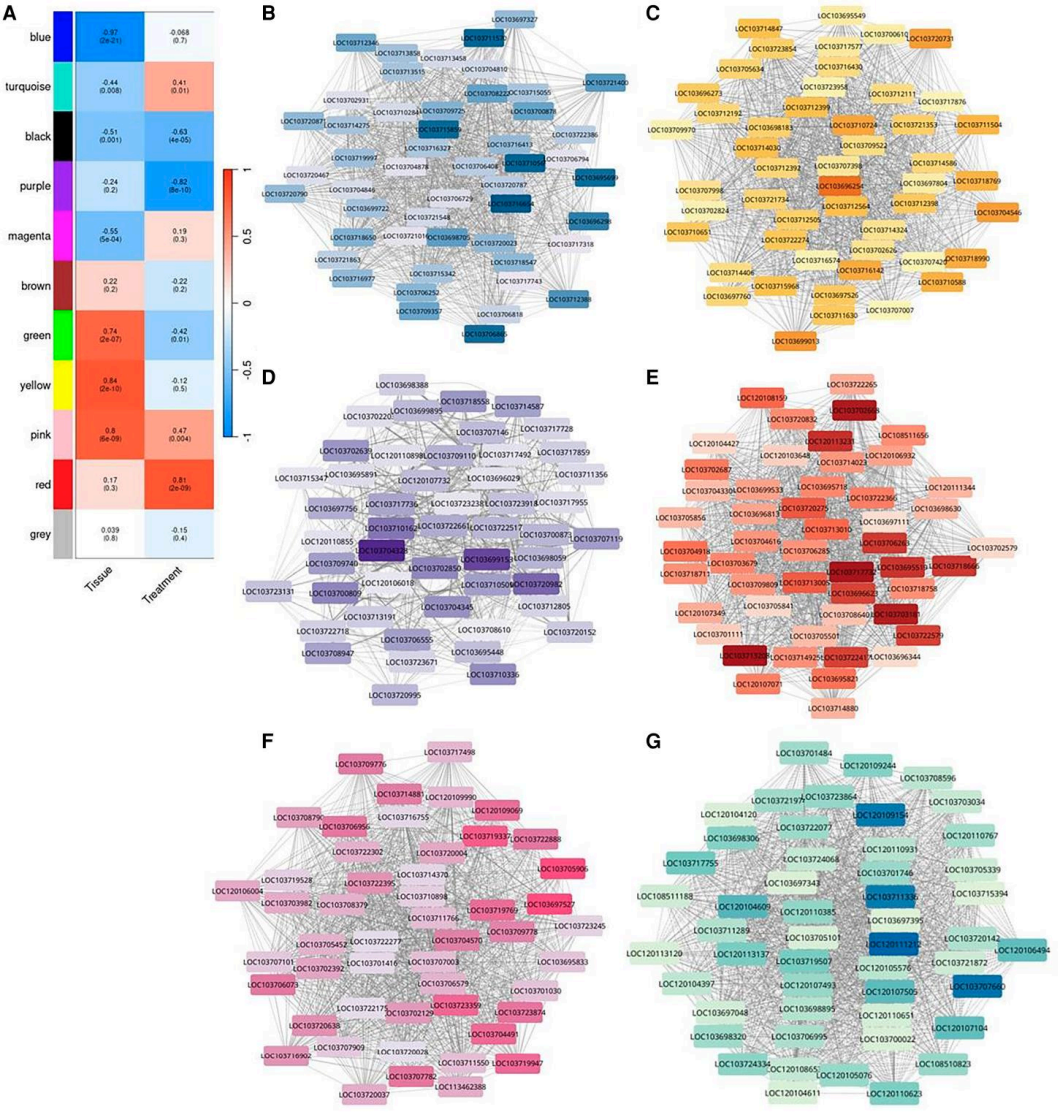
Weighted gene co-expression network analysis-identified modules correlated with tissue type and infection. (A) Heatmap showing the correlation (Pearson’s *r*) of 11 gene module eigengenes with the traits Tissue (Shoot vs. Root) and Treatment (Infected vs. Control). Red indicates a positive correlation, blue a negative correlation. (B–G) Network visualization of the top 50 hub genes (based on module membership, kME) within six selected modules: (B) negatively correlated with shoot tissue, representing a root-specific expression profile (blue); (C) positively correlated with shoot tissue, enriched for photosynthetic genes (yellow); (D) negatively correlated with infection, containing genes suppressed by *F. proliferatum* (purple); (E) positively correlated with infection, enriched for defense-related genes (red); (F) positively correlated with both shoot tissue and infection, representing a shoot-specific defense response (pink); and (G) positively correlated with infection and negatively with shoot tissue, representing a root-specific defense response (turquoise). Node color intensity within networks corresponds to the strength of the kME value.

The analysis revealed 11 distinct co-expression modules; however, the grey module representing genes that did not correlate well with any cluster was excluded from downstream interpretation. This left in 10 biologically meaningful modules for further investigation (Fig. 9A).

Among these, the blue exhibited a strong negative correlation with tissue type (*r*=−0.97, *P*=2×10^−21^), including root-specific expression, as root samples were coded as 0 and shoot as 1 (Fig. 9B). Conversely, the yellow module showed a strong positive correlation with tissues (*r*=0.84, *P*=2×10^−10^), pointing to shoot-specific gene expression (Fig. 9C).

The purple module was strongly negatively correlated with treatment (*r*=−0.82, *P*=8×10^−10^), suggesting that genes in this module were significantly down-regulated upon infection across tissues and time points (Fig. 9D). In contrast, the red module showed a positive correlation with treatment (*r*=0.81, *P*=2×10^−9^), indicating that its constituent genes were broadly up-regulated in response to *Fp* infection (Fig. 9E).Interestingly, the pink module was positively correlated with both tissues (*r*=0.8, *P*=6×10^−9^) and treatment (*r*=0.47, *P*=0.004), implying that genes in this module are predominantly expressed in shoot tissue and further induced by infection highlighting a shoot-specific defense response (Fig. 9F).

The turquoise module showed a moderate negative correlation with tissue (*r*=−0.44, *P*=0.008) and a positive correlation with treatment (*r*=0.41, *P*=0.01), suggesting a root-specific transcriptional response to infection, similar in pattern to the pink module but localized in roots (Fig. 9G).

### qRT-PCR validation

The robustness of the RNA-seq data was confirmed by qRT-PCR validation of selected DEGs. Expression trends from qRT-PCR showed high concordance with RNA-seq analysis for genes involved in defense and hormone signaling (Supplementary Fig. S4).

**Fig. 10.**
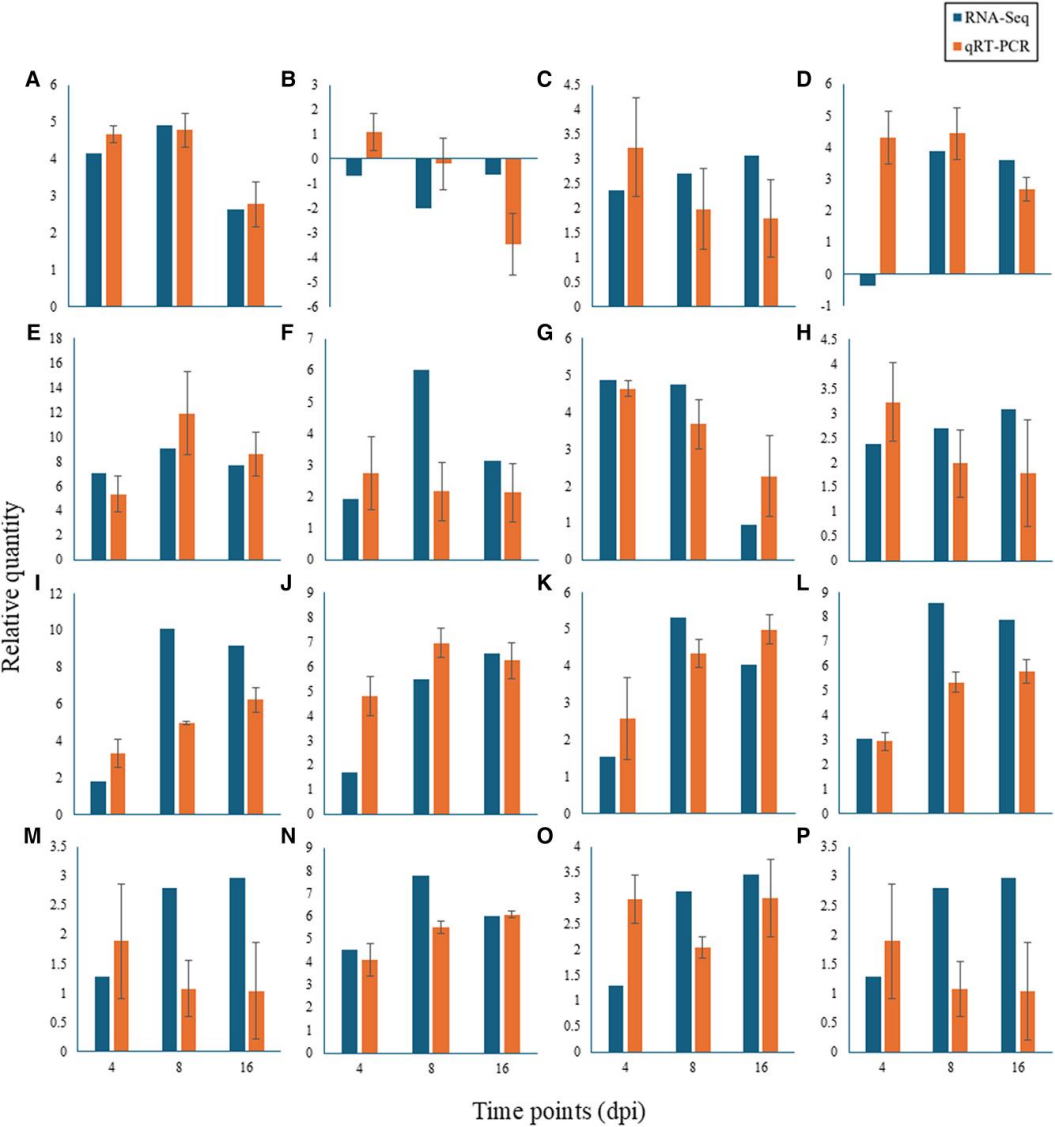
Validation of key defense gene expression by qRT-PCR confirms RNA-seq data and reveals tissue-specific dynamics. Expression profiles of selected defense-related genes in root (A–H) and shoot (I–P) tissues of date palm at 4, 8, and 16 dpi with *Fusarium proliferatum* DSM106835. Gene expression in root tissues includes (A) *TIFY 9-like* (*LOC103696392*), (B) *jasmonoyl-L-amino acid synthetase GH3.5-like* (*LOC103716872*), (C) *WRKY transcription factor 22-like* (*LOC103709031*), (D) *WRKY transcription factor WRKY24* (*LOC103708865*), (E) *pathogenesis-related protein PRB1-3-like* (*LOC103712270*), (F) *ethylene-insensitive protein 3* (*LOC103709120*), (G) *ethylene-response factor C3-like* (*LOC103703576*), and (H) *1-aminocyclopropane-1-carboxylate synthase-like* (*LOC103699314*). Gene expression in shoot tissues includes (I) *1-aminocyclopropane-1-carboxylate synthase-like* (*LOC103699314*), (J) *1-aminocyclopropane-1-carboxylate synthase 7* (*LOC103709553*), (K) *ethylene-response factor C3-like* (*LOC103703576*), (L) *TIFY 9-like* (*LOC103696392*), (M) *jasmonoyl-L-amino acid synthetase GH3.5-like* (*LOC103716872*), (N) *pathogenesis-related protein PRB1-3-like* (*LOC103712270*), (O) *WRKY transcription factor WRKY24* (*LOC103708865*), and (P) *mitogen-activated protein kinase kinase 5* (*LOC103718404*). Validated genes are involved in JA (A, B, L, M), ET (F, G, H, I, J, K), and SA (E, N) signaling, as well as the MAPK cascade (P) and PR responses (E, N). Gene expression levels from RNA-seq (blue, log_2_ fold change) and qRT-PCR (orange bars, relative expression calculated by the method where *actin-2* (*LOC103720863*) was used as a reference gene) are shown. dpi, days post-inoculation; ET, ethylene; JA, jasmonic acid; MAPK, mitogen-activated protein kinase; PR, pathogenesis-related; SA, salicylic acid.

In root tissues, key validated genes included the JA signaling repressor, *TIFY 9-like* (*LOC103696392*), which was up-regulated (Fig. 10A), while the JA-isoleucine (JA-Ile) conjugate-producing enzyme *jasmonoyl-L-amino acid synthetase GH3.5-like* (*LOC103716872*) briefly increased at 4 dpi, most likely as a result of injury, and then decreased at 8 and 16 dpi (Fig. 10B). There was a significant up-regulation of *WRKY22-like* (*LOC103709031*), a transcription factor that is normally activated by PAMPs and downstream of MAPK signaling (Fig. 10C). Although *WRKY24* (*LOC103708865*), another important transcription factor linked to phytoalexin production and pathogenesis-related (PR) gene activation, was up-regulated, its expression gradually decreased throughout the course of the infection (Fig. 10D). A crucial indicator of plant defense, the PR protein *PRB1-3-like* (*LOC103712270*), was substantially up-regulated, and its expression gradually increased over time (Fig. 10E). In the ET pathway, the crucial nuclear transcription factor *EIN3* (*LOC103709120*) was continuously up-regulated, suggesting ongoing ET signaling activity (Fig. 10F). Although its expression decreased from 4 to 16 dpi, the transcription factor *ERF* (*LOC103703576*), a direct target of EIN3, was likewise up-regulated (Fig. 10G). The *1-aminocyclopropane-1-carboxylate synthase-like* gene (*LOC103699314*), encoding the rate-limiting enzyme in ET biosynthesis, was also up-regulated in root tissue with its expression decreasing over time at 4, 8, and 16 dpi, respectively (Fig. 10H). These profiles demonstrate how important the JA, ET, and WRKY transcription factors are for root-based immunity.

In shoot tissues, the defense response showed distinct dynamics. The ET biosynthesis gene *ACS* (*LOC103699314*) and its isoform *ACS7* (*LOC103709553*) were up-regulated, along with *ERF* (*LOC103703576*) (Fig. 10I–K). The JA signaling repressor *TIFY 9-like* (*LOC103696392*) and the biosynthetic enzyme *GH3.5-like* (*LOC103716872*) were significantly up-regulated in roots (Fig. 10L, M). The PR protein *PRB1-3-like* (*LOC103712270*) and *WRKY24* (*LOC103708865*) showed sustained up-regulation, and the upstream activator *MAPKK5* (*LOC103718404*) was also induced (Fig. 10N–P). Although this correlation points toward a possible role in systemic signaling, functional studies are required to confirm causality.

## Discussion

### Validation of pathogenicity and mode of infiltration

Our study reveals the complex transcriptomic responses of date palm to *Fp* DSM106835, the causative agent of SDS. The results demonstrate a potentially coordinated defense strategy involving signaling cascades, hormonal modulation, and metabolic reprogramming. These correlations require further functional validation.

A time-course analysis at 4, 8, and 16 dpi, combined with tissue-specific profiling of roots and shoots, offered critical insights into the progression of infection and host response. Disease symptoms followed a clear timeline, with initial wilting observed at 8 dpi and more advanced manifestations, such as root shrinkage, becoming evident by 16 dpi (Fig. 1), consistent with earlier reports on SDS development (Alwahshi *et al.*, 2019).

Increasing fungal DNA levels, quantified by qRT-PCR (Fig. 1E), confirmed successful tissue colonization. The correlation between symptom emergence and fungal proliferation highlights the value of a time-resolved transcriptomic approach for unraveling the dynamics of date palm response to *Fp* infiltration.

Similar time-resolved RNA-seq studies in other monocots have provided comparable insights into temporal pathogen colonization and host responses. For example, in maize (*Zea mays*) infected with *F. proliferatum*, early stages were characterized by asymptomatic fungal establishment followed by a sharp increase in fungal biomass and defense gene activation (Sun *et al.*, 2024). Likewise, progressive infection patterns were observed in oil palm challenged with *Ganoderma boninense* (Dhillon *et al.*, 2021), where symptom onset correlated with marked transcriptional reprogramming in defense and stress-related genes. The disease progression observed in date palm thus mirrors the biphasic infection dynamics typical of monocot–*Fusarium* pathosystems, reinforcing the suitability of our time-point design for capturing the early, transition, and late immune phases (Thomma *et al.*, 1998; Ding *et al.*, 2011; Robles-Barrios *et al.*, 2022).

### Analysis of differentially expressed genes involved in hormone signaling

RNA-seq analysis revealed extensive reprogramming of gene expression across different time points and tissues, highlighting the dynamic nature of the date palm defense response to *Fp* infection (Fig. 2). Notably, a higher number of DEGs were detected in roots than shoots, reflecting a more immediate and robust response at the primary infection site. This is consistent with the direct contact between the pathogen and root tissues, which rapidly activates defense-related gene expressions. Early root responses were enriched in defense-related GO terms, such as ‘response to chemical’, and ‘cell wall organization’ at 4 dpi (Fig. 3). These patterns are consistent with early pathogen recognition and possible structural reinforcement to contain infection (Boller and Felix, 2009). By 16 dpi, enriched GO terms shifted toward those associated with proteolysis, possibly reflecting a transition from active defense to cellular maintenance and repair under prolonged stress.

In contrast, early shoot enrichment in photosynthesis-related GO terms likely reflects systemic stress responses or the impact of fungal toxins (Fig. 3). The transient activation of photosynthetic pathways may represent an adaptive strategy whereby shoots enhance energy production to support defense-related processes and maintain plant resilience. However, this enrichment also suggests a potential disruption of photosynthetic function, possibly induced by fungal toxin activity commonly associated with necrotrophic pathogens (Aucique-Pérez *et al.*, 2019) or by resource reallocation toward defense responses (Huot *et al.*, 2014). At later infection stages, the concurrent enrichment of ABA-related GO terms alongside photosynthesis-related terms indicates the involvement of ABA in systemic signaling and stress adaptation. As such, these associations remain correlative, and functional validation will be necessary to confirm the specific role of ABA in systemic defense (García-Andrade *et al.*, 2011).

Our hormone-related DEG profiles are broadly consistent with other monocot transcriptome studies. In the maize–*F. proliferatum* interaction, strong activation of JA/ET biosynthesis genes was reported alongside suppression of auxin signaling (Sun *et al.*, 2024), indicating the antagonistic hormonal crosstalk identified in our dataset. Similarly, in bananas infected by *Fusarium oxysporum* f. sp. *cubense*, early ABA signaling and later JA/ET activation were shown to modulate the transition from biotrophic to necrotrophic phases of infection (Li *et al.*, 2012). However, unlike these annual monocots, date palm demonstrated a sustained up-regulation of ABA and proteolytic signaling at 16 dpi, reflecting a long-term stress-adaptive phase likely tied to its perennial nature. This distinction suggests that hormone crosstalk in date palm underpins not only defense but also resilience mechanisms associated with prolonged pathogen pressure.

### Pathways induced in response to *Fusarium proliferatum* infection

KEGG pathway enrichment analysis provided valuable insights into the major signaling pathways activated in date palm during *Fp* infection. Notably, the MAPK signaling pathway was significantly up-regulated (Figs 4, 5A) highlighting its central role in mediating defense responses against fungal pathogens.

In parallel, the plant hormone signal transduction pathway was also enriched (Figs 4, 6, 7), reflecting the intricate hormonal crosstalk among SA, JA, ET, and ABA in orchestrating immune responses (Robert-Seilaniantz *et al.*, 2011). The concurrent activation of both SA- and JA/ET-mediated pathways suggests that date palm mounts a multilayered immune response, likely tailored to the hemi-biotrophic nature of *Fp* (Glazebrook, 2005).

In addition, the phenylpropanoid biosynthesis pathway (Fig. 4) was significantly up-regulated, emphasizing its contribution to the production of phenolic compounds with antifungal properties. The induction of this pathway leads to the accumulation of defense-related secondary metabolites. Of particular importance is phenylalanine ammonia-lyase, which catalyses the conversion of phenylalanine to cinnamic acid, the precursor of a wide array of phenylpropanoid derivatives involved in structural reinforcement and pathogen resistance (Chen *et al.*, 2024). This metabolic enhancement reinforces the overall defense strategy employed by date palm against *Fp* infection.

The enrichment of MAPK signaling, plant–pathogen interaction, and phenylpropanoid biosynthesis pathways aligns closely with patterns reported in other monocots under fungal attack. For instance, both *Fusarium*–maize and *Fusarium*–wheat transcriptomes revealed up-regulation of MAPK cascade genes, WRKY transcription factors, and phenylpropanoid enzymes (Ngou *et al.*, 2022; Sun *et al.*, 2024). In oil palm–*Ganoderma* infection, activation of similar pathways was also accompanied by induction of lignin biosynthesis genes linked to cell wall fortification (Dhillon *et al.*, 2021). These parallels suggest a conserved defense architecture among monocots, although the sustained proteasome and ABA pathway activation unique to date palm indicates an extended adaptive response beyond typical short-term immunity.

### Integrating date palm defense responses into the ‘zig–zag’ model of plant immunity

Our time-resolved transcriptome analysis reveals a sophisticated, multi-layered immune response in date palm against *Fp*. These findings regarding the hormonal crosstalk and key pathway activation can be powerfully synthesized within the established ‘zig–zag’ model of plant immunity (Jones and Dangl, 2006). This framework allows us to interpret the temporal dynamics of the defense response as a sequential escalation from broad-spectrum to highly specific immunity.

The early responses observed at 4 dpi are characteristic of PTI. The significant up-regulation of genes encoding PRRs, such as *FLS2* and *CERK1*, indicates successful perception of fungal PAMPs or microbe-associated molecular patterns (Boller and Felix, 2009). This perception initiated a robust MAPK signaling cascade, leading to a ROS burst, activation of WRKY TFs (e.g. WRKY22, WRKY29), and callose deposition, recognized as hallmarks of a successful PTI response aimed at halting pathogen entry and growth (Yu *et al.*, 2024). The concurrent activation of cell wall reinforcement and phenylpropanoid pathways further represents the foundational, PTI-associated barriers.

However, the progressive colonization by *Fp*, evidenced by increasing fungal biomass and symptom severity, suggests that the pathogen can partially suppress or evade this initial PTI layer, likely through the secretion of effector proteins. This suppression represents the ‘zag’ in the model, where the pathogen gains a temporary advantage. Our data indicate the activation of the second layer of defense, ETI. This is supported by the sustained and often amplified expression of defense genes at later time points (8 and 16 dpi), particularly in roots. The pronounced up-regulation of NLR-type genes (e.g. *LOC103704328*, a putative disease resistance protein) and the hyper-induction of key TFs like WRKY33 (a central regulator of ETI responses) strongly suggest that specific *Fp* effectors are being recognized by date palm resistance (R) proteins (Ngou *et al.*, 2022). This ETI response is quantitatively stronger and more prolonged, often associated with a hypersensitive response (HR), which aligns with the observed transcriptional shift towards proteolysis and cellular reorganization at 16 dpi.

The intricate hormonal crosstalk we documented is a critical modulator of this PTI–ETI interplay (Pieterse *et al.*, 2012). The simultaneous induction of both SA and JA/ET signaling pathways reflects the plant’s attempt to mount a balanced defense against a hemi-biotrophic pathogen like *F. proliferatum*. Early JA/ET signaling may contribute to PTI and initial containment, while the later, potent ETI response is likely amplified by SA-mediated signaling, leading to the systemic and robust expression of *PR* genes we observed in both roots and shoots. This synergistic interaction between PTI and ETI, fine-tuned by hormonal networks, creates a resilient defense system where the strengths of one layer compensate for the temporary suppression of the other (Yu *et al.*, 2024).

When viewed alongside other monocot systems, the date palm immune response reflects both conservation and specialization. In maize–*F. proliferatum* interactions and early activation of PRRs such as FLS2 and downstream WRKY22/33 regulators similarly conformed to the PTI–ETI cascade (Sun *et al.*, 2024). However, date palm displayed a prolonged transcriptional continuum, with extended induction of ET, ABA, and proteolytic genes into later infection stages. This sustained defense signature, absent in annual cereals, supports the idea that perennial monocots deploy temporally layered immunity, integrating rapid pathogen recognition with delayed stress accommodation to maintain tissue integrity during chronic infection.

In conclusion, the dynamic transcriptomic reprogramming in date palm against *Fp* is not a disjointed set of responses but a coherent, escalating immune strategy that aligns with the zig–zag model. The early PTI response provides the first line of defense, which is subsequently reinforced and amplified by a more specific ETI response, with hormonal signals acting as the central orchestrators of this defense continuum. This integrated perspective highlights that breeding for durable SDS resistance should target components of both PTI (e.g. enhancing PRR diversity or MAPK signaling efficiency) and ETI (e.g. pyramiding *R* genes), while considering the master regulatory role of key TFs and hormone pathways.

### Key gene modules identified using WGCNA in response to *Fusarium proliferatum* infection

WGCNA revealed key gene modules underlying tissue-specific defense strategies in date palm during *Fp* infection. The blue module, negatively correlated with shoot identity, was enriched in root-expressed genes such as *LOC103716654* (*myosin-1-like*), *LOC103710569* (*LRR receptor-like kinase*), and *LOC103706865* (*PBL7*), which are involved in cytoskeletal organization, pathogen recognition, and immune signaling, respectively. These genes highlight the pivotal role of roots in environmental sensing and initiating localized defense responses (Kumar *et al.*, 2020; Tao *et al.*, 2024).

In contrast, the yellow module, positively correlated with shoots, contained genes linked to chloroplast function and photosynthesis, including *LOC103696254* (*leucine-tRNA ligase*) and *LOC103699013* (*glucose-6-phosphate dehydrogenase*), reflecting robust metabolic activity in healthy shoots and its impairment during infection (Hong *et al.*, 2012; Agudelo-Romero *et al.*, 2015).

Modules responsive to infection further elucidated hub genes central to defense. The purple module, strongly negatively correlated with treatment (*r*=−0.82), included *LOC103704328* (putative disease resistance protein *RGA3*), whose suppression may indicate pathogen-induced immune invasion, similar to patterns observed in cassava root rot (Hohenfeld *et al.*, 2024). Although not yet functionally characterized, *LOC103720982* (zinc finger BED domain-containing protein; *RICESLEEPER 2-like*) may serve a regulatory role in stress adaptation and warrants further investigation (He *et al.*, 2019).

Conversely, the red module was positively correlated with infection and enriched in defense-related genes such as *LOC103713208* (*monothiol glutaredoxin-S9-like*), *LOC103702668* (subtilisin-like protease *SBT3.11*), and *LOC103706805* (*U-box domain-containing protein 12-like*). These genes participate in redox regulation, proteolytic activation of defense peptides, and ubiquitin-mediated signaling (hallmarks of PTI and ETI responses) (Couturier *et al.*, 2014; Xu *et al.*, 2022).

Tissue-specific defense was further delineated by the pink and turquoise modules. The pink module encompassed shoot-specific stress-responsive genes like *LOC103705906* (*ethanolamine-phosphate cytidylyltransferase-like*) and *LOC103697303* (chaperone protein *ClpD2*), indicating systemic signaling and chloroplast damage mitigation (Ramundo *et al.*, 2014; Xing *et al.*, 2022). Meanwhile, the turquoise module, representing root-specific responses, included *LOC120111212* (*ARR18-like response regulator*) and *LOC103707660* (*AGL29 MADS-box protein*), implicated in hormonal regulation and transcriptional control (Veerabagu *et al.*, 2012; Quesada-Traver *et al.*, 2022), along with *LOC103703631* (*pectinesterase-like*) and *LOC103702668* (*SBT3.11*), associated with cell wall remodeling and immune activation (Fernández *et al.*, 2012; Kumaravel *et al.*, 2020). Together, these co-expression modules unveil a coordinated, tissue-specific defense strategy in date palm, where roots mount strong local responses and shoots engage in systemic signaling and stress adaptation. This integrative network analysis offers novel insights into how date palms manage defense while maintaining tissue function during SDS progression.

Comparative co-expression analyses in other monocots have rarely explored tissue-specific defense networks, making this study particularly novel. While root-associated modules enriched in receptor-like kinases and WRKYs are consistent with maize and wheat root transcriptomes during *Fusarium* infection (Ngou *et al.*, 2022; Sun *et al.*, 2024), our identification of shoot-specific modules linked to chloroplast and stress-mitigation genes parallels findings in oil palm–*Ganoderma* systems (Dhillon *et al.*, 2021). The integration of WGCNA thus reveals a conserved modular defense framework across monocots but also underscores the palm-specific strategy of maintaining photosynthetic and metabolic balance during sustained infection. This system-level insight advances the understanding of transcriptome dynamics in long-lived monocots and provides a foundation for identifying durable resistance genes in date palm.

It is important to note that the present study is based on transcriptomic correlations. While these data provide valuable hypotheses and candidate genes, functional assays (e.g. gene silencing, overexpression, or mutant analysis) are of top priority to establish causal links between specific DEGs and resistance, as well as to confirm systemic signaling mechanisms.

## Supplementary Material

eraf540_Supplementary_Data

## Data Availability

The raw RNA-seq data generated in this study, as well as count matrix and DESEq2 normalized read counts, have been deposited in the NCBI Sequence Read Archive (SRA) under the Bioproject PRJNA1233823.

## Supplementary data

The following supplementary data are available at *JXB* online.

Fig. S1. Quality control and statistical analysis for differential expressions.

Fig. S2. Principal component analysis and differential gene expression analysis of tissue and disease effects.

Fig. S3. Volcano plots for differentially expressed genes in root and shoot samples upon inoculation with *Fusarium proliferatum* DSM106835.

Fig. S4. Validation of RNA-seq data by qRT-PCR analysis of selected DEGs in *Fp* DSM106835-infected date palm seedlings.

Table S1. List of qRT-PCR primers (sequence 5′ to 3′) used in the study.

Table S2. The count data from mapping all samples against the reference genome of date palm.

Table S3. Deseq2 results for each sample, showing comparisons for roots samples at 4, 8, and 16 dpi, as well as shoot samples at 4, 8, and 16 dpi.

Table S4. List of KEGG pathways enriched in root tissues of date palm seedlings during *Fp* infection at 4, 8, and 16 dpi.

Table S5. List of KEGG pathways enriched in shoot tissues of date palm seedlings during *Fp* infection at 4, 8, and 16 dpi.

Table S6. List of DEGs from the MAPK signaling pathway (KEGG: 04016) and plant–pathogen interaction pathway (KEGG: pda04626) across all tissues and all time points in date palm seedlings during *Fp* infection.

Table S7. List of DEGs from the cystine and methionine metabolism pathway (pda00270), α-linolenic acid metabolism pathway (pda00592), phenylalanine metabolism (pda00360), tryptophan metabolism (pda00380), and carotenoid biosynthesis pathway (pda00906) across all tissues and time points in date palm seedlings during *Fp* infection.

Table S8. List of DEGs from the plant hormone signal transduction pathway (pda04075) across all tissues and time points in date palm seedlings during *Fp* infection.

Table S9. List of DEGs encoding kinase proteins across all tissues and time points in date palm seedlings during *Fp* infection.

Table S10. List of DEGs encoding transcription factors in roots and shoots of date palm seedlings during *Fp* infection at 4, 8, and 16 dpi.
