# Supplementary material for: Time-resolved transcriptome analysis reveals molecular signatures of Fusarium proliferatum DSM106835-induced sudden decline syndrome in date palm (Phoenix dactylifera L.)
Source: J Exp Bot. 2025 Dec 16;77(6):1676–96. doi: 10.1093/jxb/eraf540 (PMC13017883; doi:10.1093/jxb/eraf540)
Supplement: eraf540_Supplementary_Data [file eraf540_supplementary_data.zip › jexbot316454-file001.pdf]

Supplementary Figures

Time-resolved Transcriptome Analysis Reveals Molecular Signatures of *Fusarium proliferatum* DSM106835-induced sudden decline syndrome in Date Palm (*Phoenix dactylifera* L.)

Gouthaman P. Purayil, Khaled A. El-Tarabily, Frank M. You and Synan F. AbuQamar*

*Correspondence: sabuqamar@uaeu.ac.ac

Supplementary Figures

Fig. S1. Quality control and statistical analysis for differential expression.

Fig. S2. Principal component analysis and differential gene expression analysis of tissue and disease effects.

Fig. S3. Volcano plots for differentially expressed genes in root and shoot samples upon inoculation with *Fusarium proliferatum* DSM106835.

Fig. S4. Validation of RNA-Seq data by qRT-PCR analysis of selected DEGs in *Fusarium proliferatum* DSM106835-infected date palm seedlings.

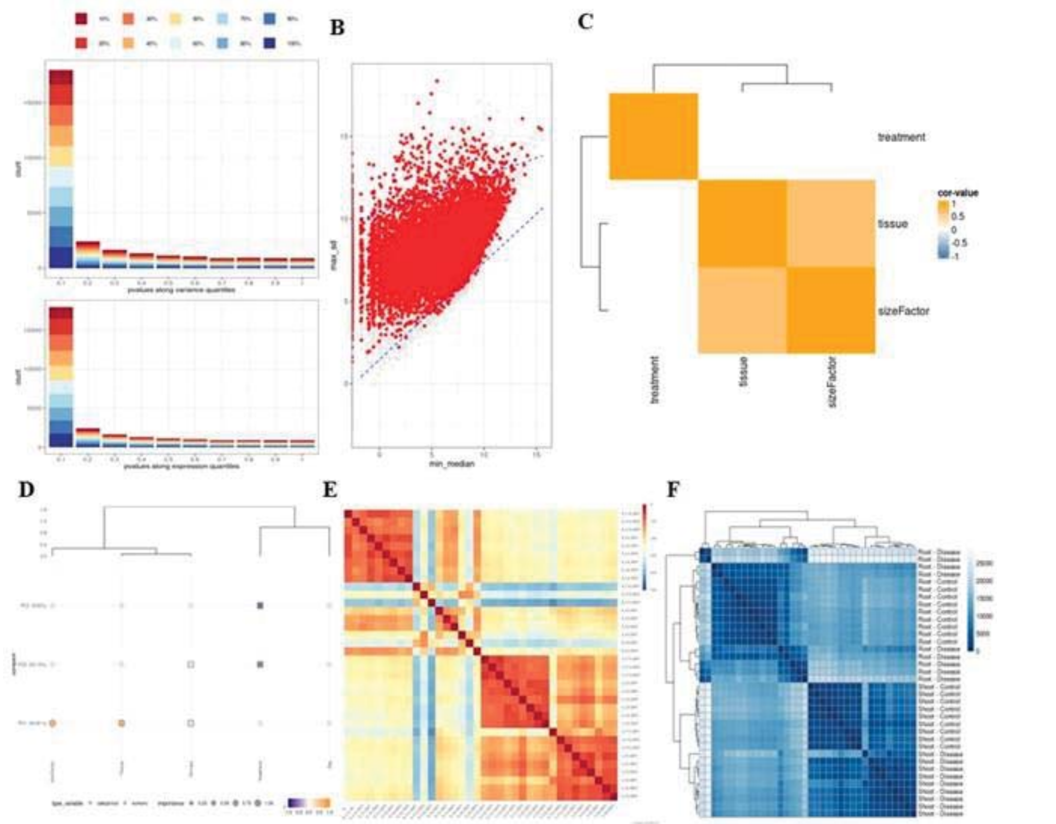

Fig. S1. Quality control and statistical analysis for differential expression. **A** Mean-variance QC plot for p-value along variance quantiles (top) and p-value along expression quantiles (bottom) tissues. **B** Scatter plot of max_sd vs. min_median. **C** Covariates correlation heatmap. **D** Covariates' effect on count data. **E** Sample-to-sample distance heatmap. **F)** Poisson distance heatmap of sample distances calculated using the. The rows and columns both represent sample names. In **A** histograms show *p*-values across variance and expression quantiles. The color gradient represents genes counts, with red indicating higher counts. In **B** plot determines the relationship between the maximum standard deviation and the minimum median gene expression across samples, with significant genes marked in red. In **C** correlation matrix with hierarchical clustering of covariates (treatment, tissue, sizeFactor and time) based on RNA-Seq data correlations. The color scale ranges from 1 (orange; positive) to -1 (blue; negative). In **D** PCA illustrates the influence of covariates on the first three principal components (PC1, PC2, PC3). In **E** Euclidean distance heatmap shows based sample relationships based on gene expression. Red/orange indicates closer similarity, and yellow/blue indicates greater dissimilarity. In **F** sample distance matrix based on Poisson Distance. R, root; S, shoot; C, control, F, *F. proliferatum*, dpi, days post-inoculation. REP, replicate; QC, quality control; PCA, principal component analysis.

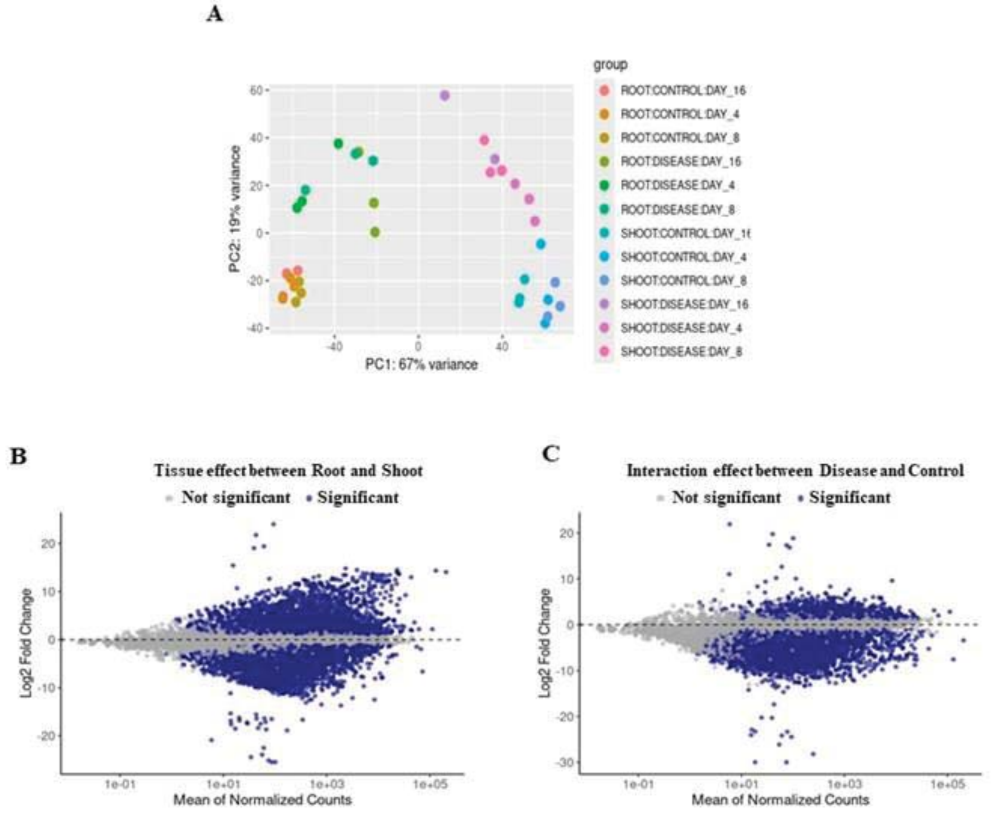

Fig. S2. Principal component analysis and differential gene expression analysis of tissue and disease effects. **A** PCA of RNA-seq samples after variance stabilizing transformation. **B** MA plot of tissue effects (root vs. shoot), displaying log2 fold changes in gene expression between shoot and root tissues. **C** MA plot of treatment effects (disease vs. control), comparing gene expression between control and disease conditions. In **A**, principal components PC1 and PC2, which account for 67% and 19% of the variance, respectively, are plotted on the x and y axes. The plot shows a clear separation between groups based on tissue and treatment. In **B**, the x-axis shows the mean of normalized counts, and the y-axis represents the log2 fold change in gene expression. Many genes display significant expression differences between tissues. In **C**, the x-axis shows the mean of normalized counts, while the y-axis represents the log2 fold change in gene expression, revealing fewer significant differences compared to tissue effects. PCA, principal component analysis.

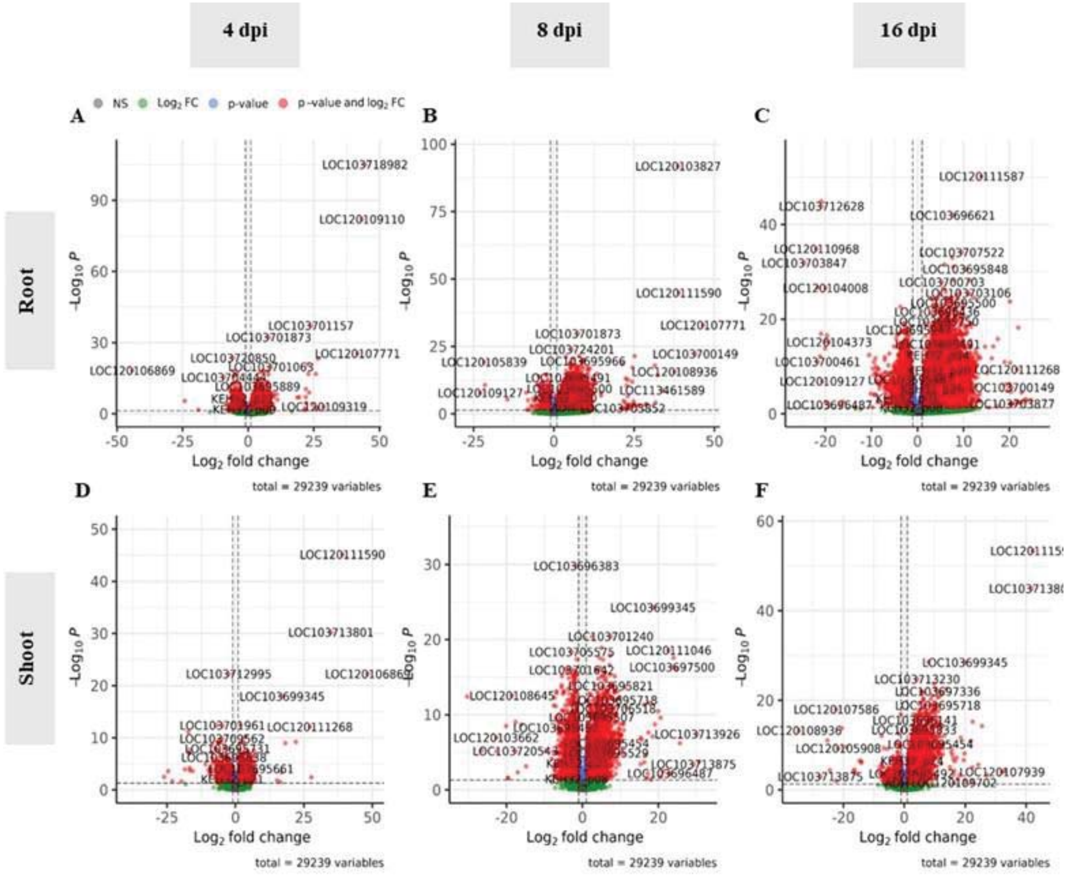

Fig. S3. Volcano plots for differentially expressed genes in root and shoot samples upon inoculation with *Fusarium proliferatum* DSM106835. **A**-**C** Root and **D**-**F** shoot samples at **A** & **D** 4 dpi, **B** & **E** 8 dpi, and **C** & **F** 16 dpi. dpi, days post inoculation.

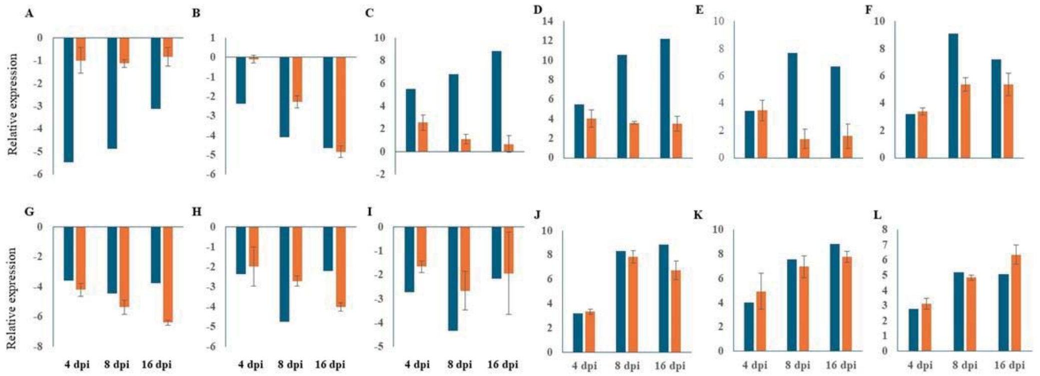

Fig. S4. Validation of RNA-Seq data by qRT-PCR analysis of selected DEGs in *Fusarium proliferatum* DSM106835-infected date palm seedlings. In root tissue, **A** *PELPK1-like* protein (LOC120104890), **B** *CASPARIAN STRIP INTEGRITY FACTOR 1-like* protein (LOC120113307), **C** disease resistance protein *RGA2-like* (LOC103706311), **D** *phylloplanin-like* protein (LOC120107305), and **E** putative disease resistance protein *RGA4* (LOC108511758) were validated. In shoot tissue, **F** *DMP3-like* protein (LOC120109005), **G** *SNF1-related protein kinase regulatory subunit beta-2-like* (LOC120111125), **H** *wee1-like protein kinase* (LOC120103898), **I** *subtilisin-like protease SBT1.7* (LOC120104047), **J** *pectinesterase-like* (LOC120107734), **K** putative *12-oxophytodienoate reductase* (LOC108510666), and **L** *chitinase 1-like* (LOC120104412). DEGs from both root and shoot tissues were randomly selected and analyzed by qRT-PCR at 4, 8, and 16 dpi with *Fp*. Blue and orange bars represent RNA-seq data ($\log_2$ FC) and qRT-PCR data ($2^{-\Delta\Delta Ct}$). *Actin-2* (LOC103720863) was used as the reference gene. DEGs, differentially expressed genes; dpi, days post-inoculation; FC, fold change.
